# Responsiveness of epigenetic aging biomarkers to longevity interventions in humans

**DOI:** 10.1038/s41591-026-04562-9

**Published:** 2026-08-21

**Authors:** Raghav Sehgal, Daniel Borrus, Jenel F. Armstrong, John Gonzalez, Jessica Kasamoto, Yaroslav Markov, Ahana Priyanka, Ryan Smith, Natàlia Carreras-Gallo, Jessica Lasky-Su, Varun B. Dwaraka, Michael J. Corley, Albert Higgins-Chen

**Affiliations:** 1 Department of Psychiatry, Yale University School of Medicine, New Haven, CT USA; 2 Program in Computational Biology and Bioinformatics, Yale University, New Haven, CT USA; 3 Program in Pathology, Yale University School of Medicine, New Haven, CT USA; 4 TruDiagnostic, Lexington, KY USA; 5 Channing Division of Network Medicine, Department of Medicine, Brigham and Women’s Hospital and Harvard Medical School, Boston, MA USA; 6 University of California, San Diego, La Jolla, CA USA

**Keywords:** Biomarkers, Data integration, Research data, Biomarkers, Geriatrics

## Abstract

Aging biomarkers can potentially allow researchers to rapidly monitor the impact of an aging intervention without the need for decade-spanning trials. However, before the use of aging biomarkers, such as epigenetic clocks, as surrogate endpoints, their responsiveness to interventions that target aging must be tested. Here we curate TranslAGE, a harmonized database of 51 public and private longitudinal interventional studies, and calculate a consistent set of 16 prominent epigenetic clocks for each study, along with 94 other DNA methylation (DNAm) biomarkers that can help explain the changes observed for each clock. Using this database, we discover patterns of responsiveness across a variety of interventions and DNAm biomarkers. For example, clocks trained to predict mortality or pace of aging show the strongest responses across all interventions and show consistent results with one another; pharmacological and lifestyle interventions drive the strongest responses from DNAm biomarkers; and the characteristics of the study population and study duration are key factors in determining the responsiveness of DNAm biomarkers to an intervention. Moreover, clocks with multiple subscores (that is ‘explainable clocks’) provide specificity and greater mechanistic insight into the responsiveness of interventions than single-score clocks. These findings can help to design future clinical trials by guiding the choice of interventions and of specific subsets of DNAm biomarkers to minimize multiple testing, study duration, study population and sample size, with the eventual aim of uncovering DNAm biomarkers that can be used as surrogate aging endpoints.

## Abstract (web summary)

The responsiveness of 16 different epigenetic aging clocks to a broad array of pharmacological and lifestyle interventions in humans was assessed across 51 different longitudinal studies, providing insights into the potential of aging clocks to serve as reliable aging biomarkers.

## Main

The geroscience hypothesis proposes that targeting the biological processes of aging may simultaneously prevent, delay or mitigate multiple chronic diseases^1^. Given the length of human lifespan, aging biomarkers are essential for evaluating interventions within feasible clinical trial timelines^2^. Surrogate biomarkers that predict long-term outcomes from short-term measurements could substantially accelerate intervention testing, whereas discovery biomarkers provide mechanistic insight into aging biology and therapeutic targets^2,3^.

Among aging biomarkers, DNA methylation (DNAm)-based epigenetic clocks are among the most extensively studied^4–6^. Epigenetic age deviations consistently predict morbidity, mortality and other aging-related outcomes^7–11^. As numerous studies have measured DNAm, and many clocks can be calculated in a consistent manner across studies, they provide a unique opportunity for systematic comparisons across interventions.

Epigenetic clocks have evolved from first-generation chronological age predictors to second-generation biomarkers of phenotypic aging, mortality risk and pace of aging, followed by reliability-optimized clocks designed to reduce technical variability^10,12–14^. More recently, explainable DNAm biomarkers such as SystemsAge, CausalAge and OMICmAge have expanded this framework by quantifying multiple biological dimensions of aging rather than a single global score^11,15,16^. In addition to epigenetic clocks, DNAm biomarkers can estimate smoking, immune cell composition, proteins, metabolites and other physiological traits. Throughout this study, we use ‘DNAm biomarkers’ as the umbrella term and reserve ‘epigenetic clocks’ for DNAm biomarkers specifically designed to measure aging.

**Fig. 1:**
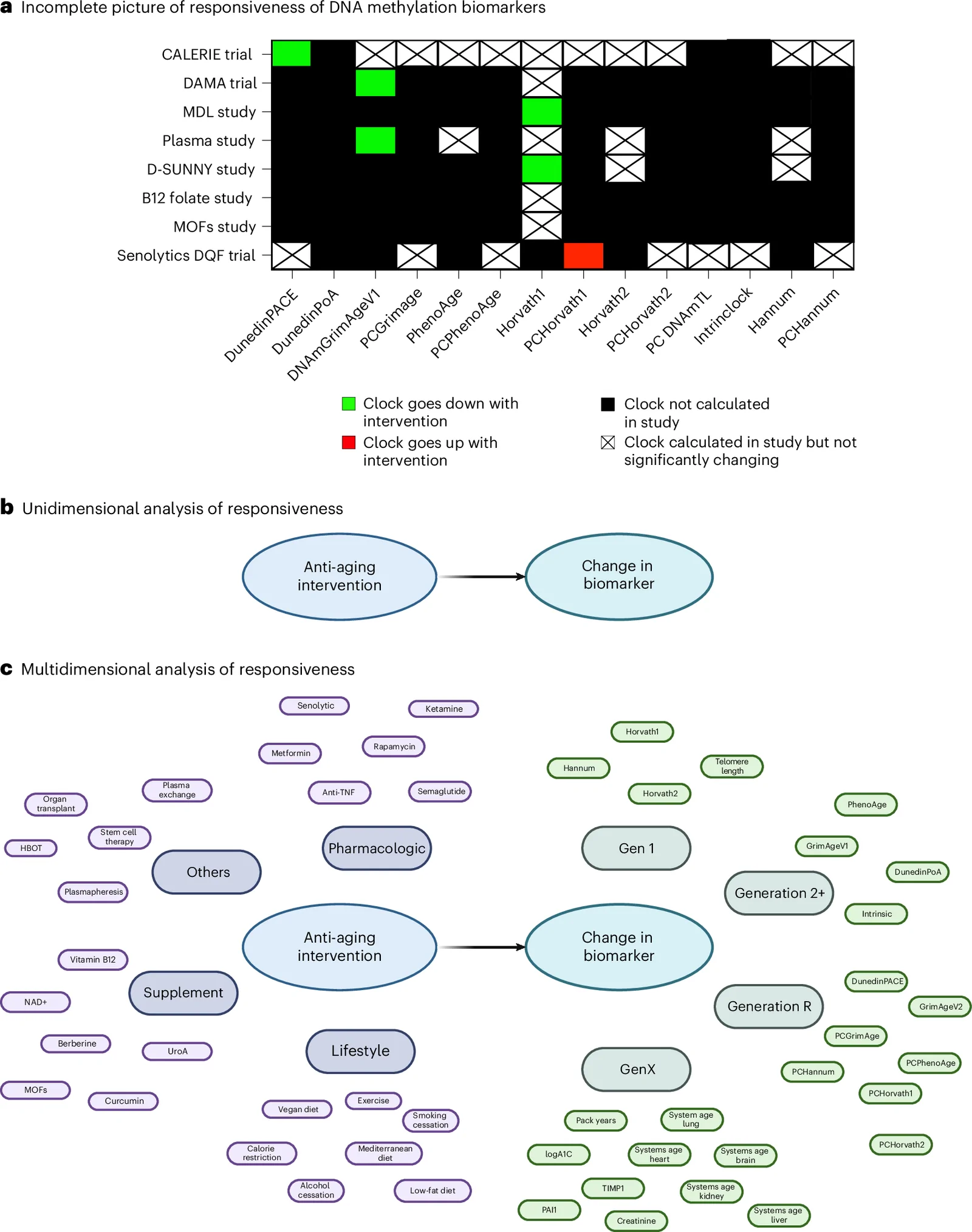
Current paradigms to study the responsiveness of DNAm biomarkers and their limitations. **a**, An incomplete picture of responsiveness: a matrix summarizes existing literature, illustrating the effects of various interventions on different DNAm biomarkers. After an intervention, DNAm-based clocks may show an increase in epigenetic age (red), a decrease in epigenetic age (green), no change (cross) or authors choose not to calculate the clock in this analysis but it was possible (black). **b**, The unidimensional analysis of responsiveness: a traditional application of the concept of responsiveness to aging biomarkers may consider only the change in one biomarker after one intervention. **c**, The multidimensional analysis of responsiveness: given that there are many aging interventions and many clocks, a complex framework is needed that categorizes anti-aging interventions and biomarkers and underscores the diverse influences of different anti-aging intervention types across different generations of DNAm biomarkers. Figure created in BioRender; Sehgal, R. https://biorender.com/k41v901 (2026).

Most validation studies have focused on biomarker associations with risk factors, morbidity and mortality^7^, whereas comparatively little is known about biomarker responsiveness to interventions, despite responsiveness being a prerequisite for surrogate endpoint qualification^17^. Existing intervention studies have examined different combinations of clocks, interventions, populations and study designs, as summarized in Fig. 1a. Fragmented literature and the calculation of inconsistent clock panels make it difficult to compare studies, identify consistently responsive biomarkers or determine whether discordant findings reflect biological differences, methodological variation, inconsistent biomarker testing or publication bias. Consequently, investigators designing new clinical trials have limited evidence to guide biomarker selection.

To overcome these limitations, we propose the integrative framework illustrated in Fig. 1c, contrasting with the fragmented approach represented in Fig. 1b. Rather than evaluating isolated combinations of interventions and biomarkers, we systematically curated and harmonized 51 human intervention studies with DNAm data, standardized study metadata and calculated a consistent panel of 16 epigenetic clocks together with 94 additional DNAm biomarkers. This unified framework enables the direct comparison of biomarker responsiveness across interventions and identifies the biomarkers and interventions showing the strongest and most reproducible responses.

## Results

### Data curation and analysis pipeline

**Fig. 2:**
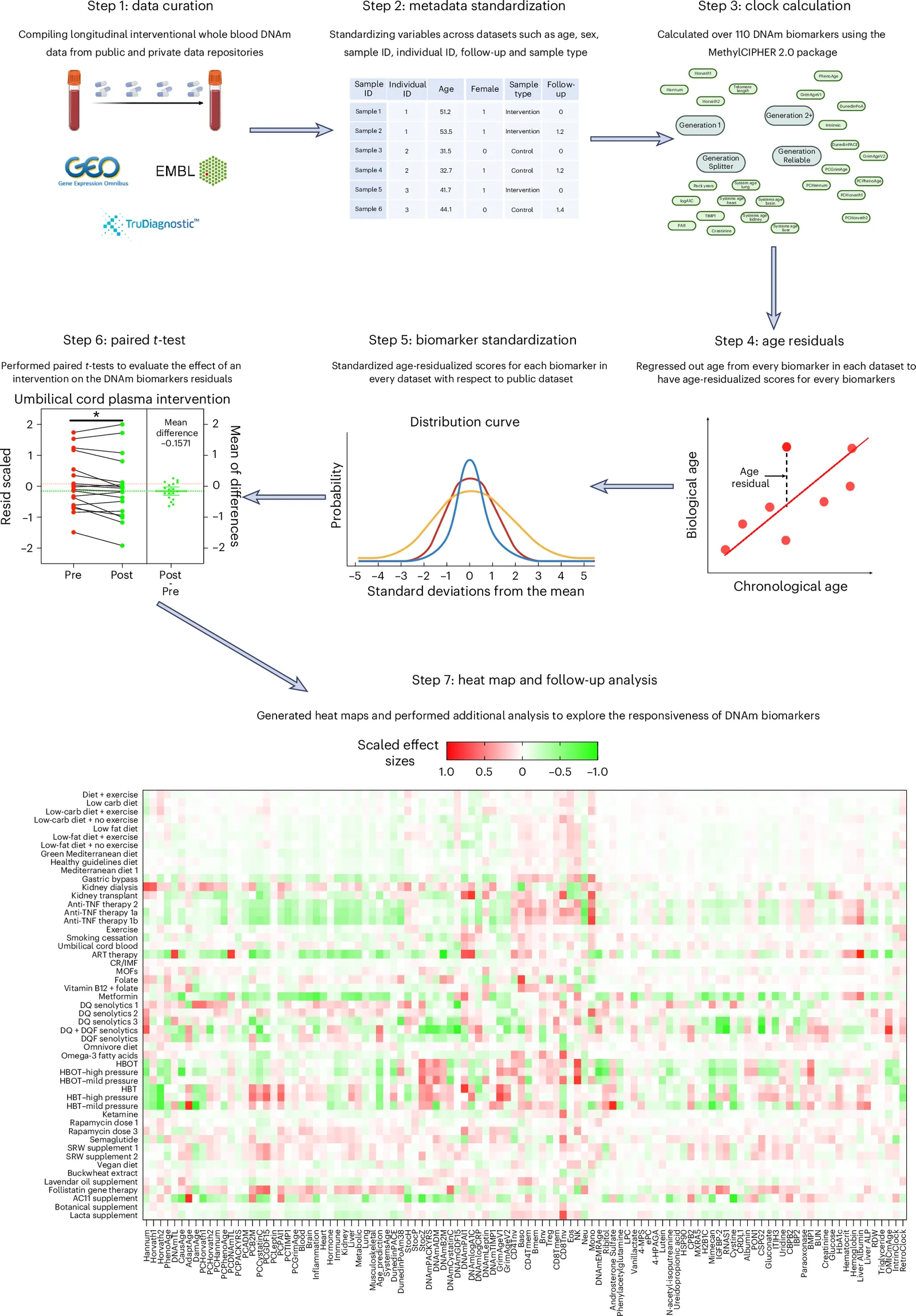
Standardized and comprehensive data curation and analysis pipeline for longitudinal interventional DNAm biomarker studies. Step 1: data curation. This initial step involved gathering a diverse array of whole blood DNAm datasets from multiple longevity-focused clinical trials and studies from various public (GEO and EMBL) and private (TruDiagnostic) repositories and standardizing their study-level metadata. Step 2: metadata standardization. Metadata for each study were harmonized to ensure consistency across datasets. Standardized variables included age, sex, sample ID, individual ID, follow-up time from baseline draw and sample type (categorizing control versus participant). Step 3: clock calculation. Using the MethylCIPHER 2.0 package, more than 110 DNAm biomarkers were calculated for each dataset. Step 4: age residuals calculation. Age was regressed out from every biomarker in each dataset to isolate age-residualized scores. Step 5: biomarker standardization age-residualized scores. For each biomarker, in every dataset, age-residualized scores were standardized (Methods) to allow comparisons across different datasets and different biomarkers. Step 6: paired *t*-test analysis. Paired *t*-tests were performed on pre- and postintervention samples to evaluate the effects of interventions on DNAm biomarkers. This statistical analysis enabled the determination of the significance of changes in biomarkers due to interventions. Step 7: heat map and follow-up analysis to explore the responsiveness of DNAm biomarkers. The heat map shows scaled effect sizes from red (positive effect) to green (negative effect), visualizing the impact of the listed interventions on the indicate DNAm biomarkers. Figure created in BioRender; Sehgal, R. https://biorender.com/4nqnt6v (2026). HBOT, hyperbaric oxygen therapy; HBT, hyperbaric therapy; ART, antiretroviral therapy; DQ, dasatinib plus quercetin; DQF, dasatinib, quercetin and fisetin.

We constructed a robust and standardized analysis pipeline for curating and analyzing a large database of interventions (Fig. 2). First, we created a list of publicly (Gene Expression Omnibus (GEO) and the European Molecular Biology Laboratory (EMBL)) and privately (TruDiagnostic) available clinical trials and studies for interventions hypothesized to promote longevity. For each study, we curated study-level metadata such as intervention type, study population, diseases in population (if any), age range and percent female. Second, we harmonized metadata in each study to have standardized columns such as: age, sex, sample ID, individual ID, follow-up time from baseline draw and sample type (control versus participant). Third, we calculated more than 110 DNAm biomarkers for each of these datasets. Fourth, we adjusted for chronological age to obtain age residuals for all DNAm biomarkers in every dataset. Fifth, we normalized the biomarker age residuals in each dataset to make them comparable across interventional studies and across biomarkers. Sixth, we performed paired *t*-test analysis to compare pre- and postintervention samples. Finally, we generated heat maps of the mean scaled effect size and performed additional analyses to understand more about the responsiveness of these biomarkers.

### Assessing longevity interventions: evidence for decreased epigenetic age across translAGE

To verify the compilation of a longevity interventions database—TranslAGE^18^—we analyzed whether 51 interventions consistently decreased epigenetic age. Our focus was on 16 prominent epigenetic clocks that were designed to be broadly applicable and not specific to any single aspect of aging. These clocks included generation 1 biomarkers (Horvath1, Horvath2 and Hannum), generation 2+ biomarkers (PhenoAge, GrimAgeV1 and DunedinPoAm38), updated clocks from earlier generations (PCHorvath1, PCHorvath2, PCHannum, PCPhenoAge, PCGrimAge, GrimAgeV2 and DunedinPACE) and generation X (Explainable) clocks developed during initial testing phases (SystemsAge, OMICmAge and DNAmEMRAge). To ensure the observed decreases in epigenetic age were genuine and not coincidental, we analyzed four control datasets and nine pro-aging event datasets (Fig. 1a and Supplementary Table 1). Two statistical analyses were performed: a one-sample *t*-test to test for deviations from zero effect size and paired *t*-tests for direct group comparisons. The results revealed that only aging events and interventions showed significant effects, with effect sizes of 0.14 (increase) and −0.06 (decrease) in the one-sample *t*-test (*P* < 0.0001). In addition, all paired *t*-test comparisons yielded significant results (*P* < 0.001).

### Certain longevity intervention categories elicit stronger responses from DNAm biomarkers

**Fig. 3:**
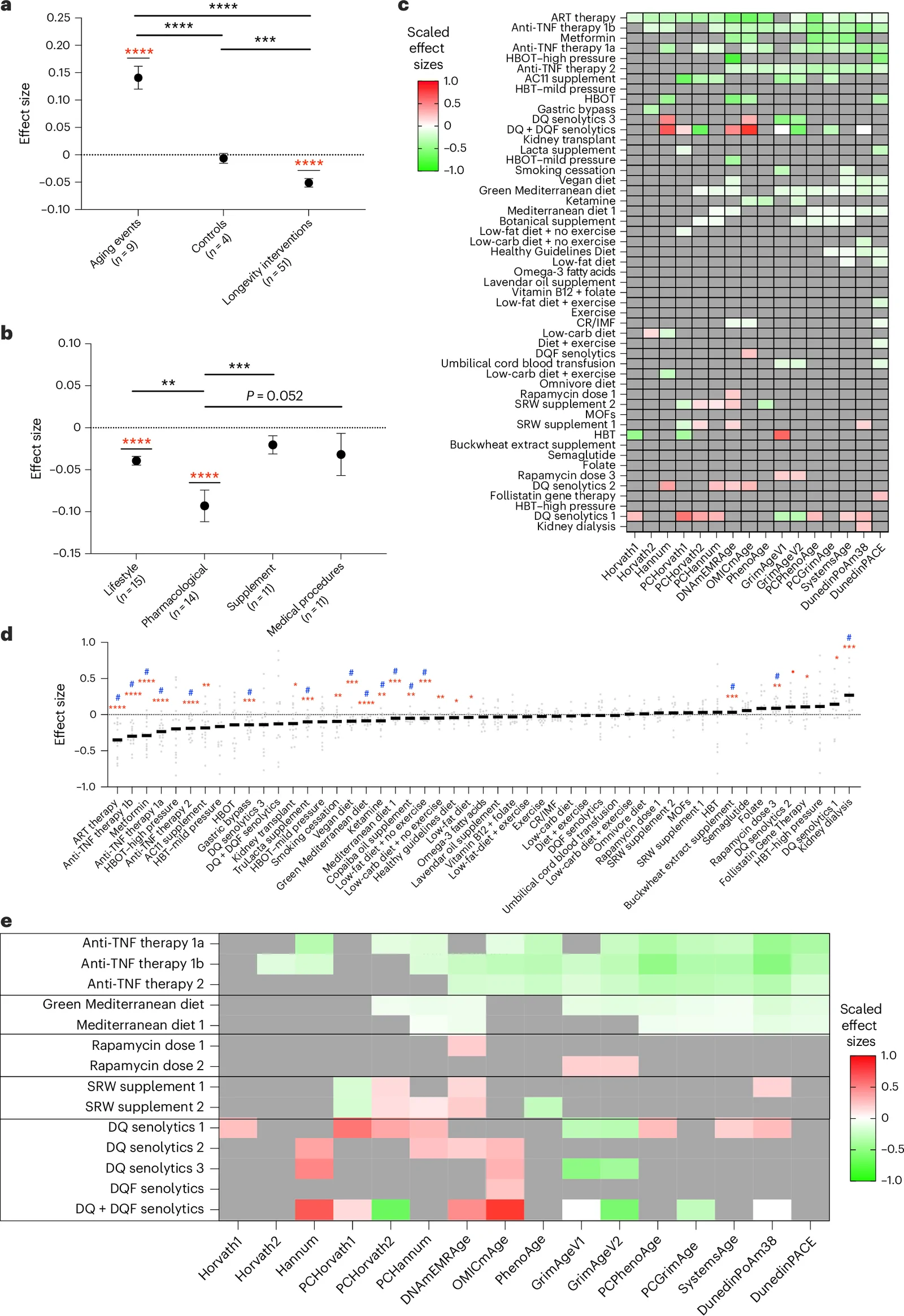
Interventions and their effects on 16 prominent DNAm biomarkers. **a**, A dot plot comparing the effect sizes for aging events, controls and longevity interventions across 16 biomarkers. (*P < 0.05, **P < 0.01, ***P < 0.001). Dotted line represents zero effect size line. Only significant *P* values are shown between different categories. **b**, A dot plot comparing the effect sizes of different intervention types across all 16 biomarkers. (*P < 0.05, **P < 0.01, ***P < 0.001). Dotted line represents zero effect size line. **c**, A heat map of biomarker responses to different interventions. The *x* axis lists the 16 biomarkers, and the *y* axis lists the interventions. Red indicates an increase in epigenetic age, green indicates a decrease in epigenetic age and gray indicates no significant effect. **d**, A plot comparing effect sizes across different interventions. The *y* axis represents the effect size, whereas the *x* axis represents different interventions (*P < 0.05, **P < 0.01, ***P < 0.001, # significant after multiple-testing corrections). The dotted line represents the zero effect size line. Only significant *P* values are shown between different categories of interventions. **e**, A heat map of interventions for which the TranslAGE database contains multiple studies of the same intervention, allowing consistency between studies to be examined. Red indicates an increase in epigenetic age, green indicates a decrease in epigenetic age and gray indicates no significant effect. HBOT, hyperbaric oxygen therapy; HBT, hyperbaric therapy; ART, antiretroviral therapy; DQ, dasatinib plus quercetin; DQF, dasatinib, quercetin and fisetin.

**Extended Data Fig. 1:**
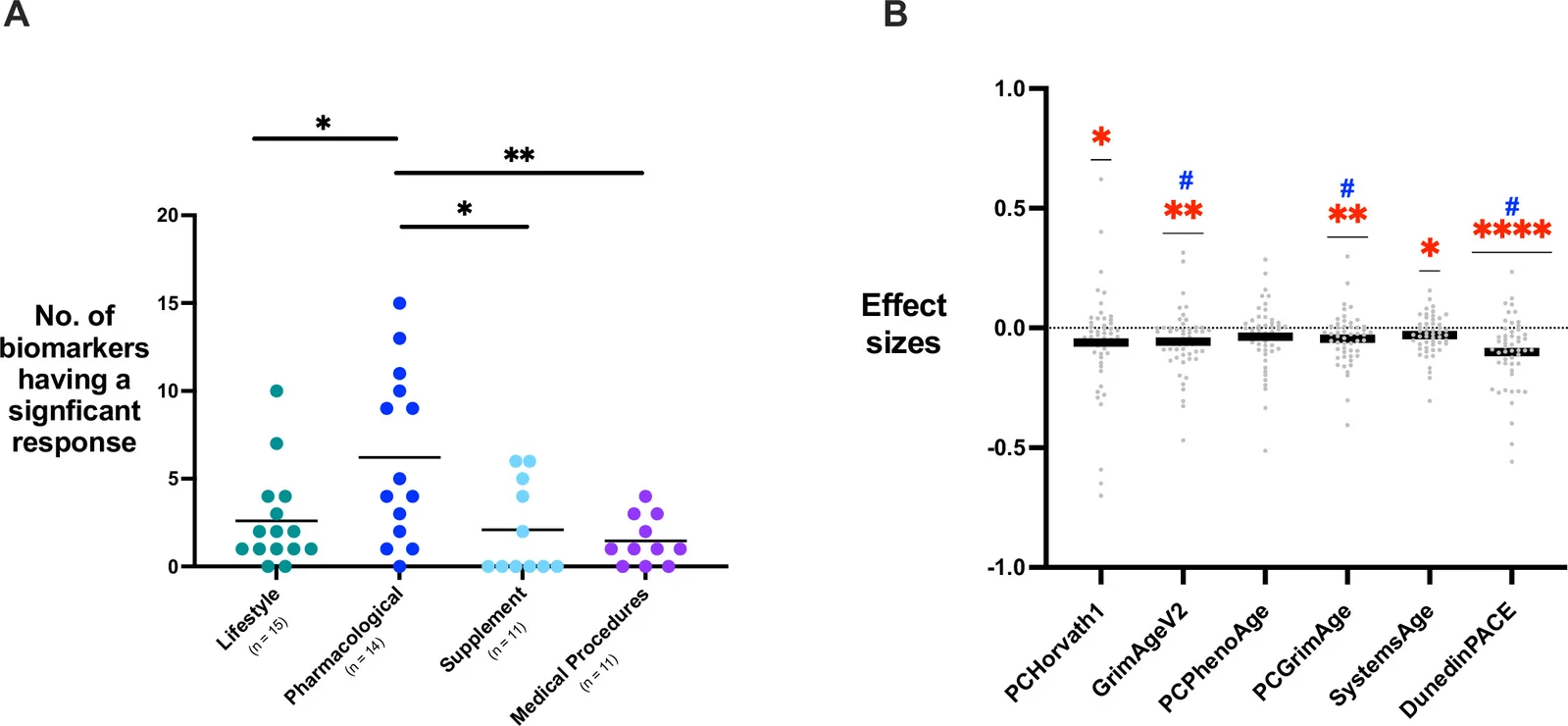
Additional analyses of intervention categories and adjustment for estimated cell fractions. **A**) Number of significant DNA methylation biomarkers responding within each intervention category. **B**) Responsiveness of technically reliable DNA methylation biomarkers after regression of estimated cell fractions, demonstrating that responsiveness is largely preserved after accounting for blood cell composition.

We categorized the interventions in our database into four groups to assess which types had greater effects on DNAm biomarkers: lifestyle (including diet and exercise), pharmacological (for example, metformin, rapamycin, semaglutide, ketamine and anti-TNF therapy), supplements (for example, omega-3 fatty acids and folate) and medical procedures (for example, hyperbaric oxygen therapy, organ transplants and gene therapy). For the first analysis, we counted the number of significant biomarkers (out of the 16 previously identified) for each intervention and plotted the results by category on a scatter plot (Extended Data Fig. 1a and Supplementary Table 2). A paired *t*-test revealed that the pharmacological category had significantly more biomarkers showing effects compared with any other category, suggesting that pharmacological interventions may elicit stronger responses from DNAm biomarkers. We further conducted a one-sample *t*-test on the effect sizes of all clocks within each intervention category (Fig. 3b and Supplementary Table 3). This analysis showed that only Pharmacological (mean effect size of −0.09307 and *t*-value of 4.948) and lifestyle interventions (mean effect size of −0.0393 and *t*-value of 7.579) significantly reduced epigenetic age, with pharmacological interventions demonstrating a larger effect. When comparing categories, only pharmacological interventions had significantly larger effect sizes compared with others (lifestyle *P* = 0.0063; supplements *P* = 0.0009; medical procedures *P* = 0.0517). Together, these findings suggest that pharmacological interventions may drive a more substantial effect on DNAm biomarkers than other types of interventions.

### Longevity interventions have varying effects on DNAm biomarkers

Finally, we plotted the effect size of all 51 interventions for the 16 DNAm biomarkers (Fig. 3c and Supplementary Table 4) with the nonsignificant effect sizes grayed out (*P* value < 0.05). Several interventions seemed to affect more DNAm biomarkers (positively or negatively) than others. To quantify this without making any initial assumptions about which biomarkers are more important, we plotted all effect sizes mapping to the 16 different biomarkers for an intervention and then performed a one-way *t*-test on effect sizes of all interventions checking for deviations from zero effect size (Fig. 3d and Supplementary Table 5). In total, 19 interventions significantly decreased epigenetic age across the 16 biomarkers (13 after multiple-testing correction), 5 significantly increased epigenetic age across the 16 biomarkers (3 after multiple-testing correction) and the remaining 26 had no significant effects.

### Consistency between biomarkers and studies supports bona fide effects of longevity interventions

Our compiled database includes a number of replicate studies of the same intervention, as well as multiple clocks that measure related constructs (for example, multiple mortality clocks). We reasoned this would be useful for determining if an intervention is indeed modifying DNAm biomarkers in a consistent manner which would support it as an effective longevity intervention. Although false positives for a given clock/intervention combination are likely to occur, it is unlikely that multiple clocks and multiple studies would repeatedly yield the same false positives. Thus we propose that interventions with bona fide effects of aging biomarkers should fulfill two rules: first, the intervention should modify DNAm biomarkers of a given generation to the same magnitude and direction in a particular study (rule 1), and a second study of the same intervention should modify the same biomarkers (rule 2). In our database, we can identify interventions that abide by both rules, one of the rules or neither of the rules (Fig. 3e). Anti-TNF therapies in patients from multiple studies (arthritis and IBD) modify nearly all generation 2+ biomarkers (both reliable and otherwise) to very similar magnitude, fulfilling both rules and supporting anti-TNF therapies as a potential method to prevent pathological aging in individuals with autoimmune disorders. Similarly, two different types of Mediterranean diets, performed in healthy cohorts, decrease similar subsets of generation 2+ DNAm biomarkers, satisfying both rule 1 and rule 2. By contrast, we see an example of two SRW supplement studies which change the same biomarkers (satisfying rule 2), but there is no consistent change in direction across those biomarkers (not satisfying rule 1—PCHorvath1 and PhenoAge decrease, whereas PCHorvath2, PCHannum and DNAmEMRAge increase). This inconsistency between biomarkers generates uncertainty about the effects of such an intervention on aging. Similarly, the five different senolytics studies have multiple biomarkers changing in different direction within a study (GrimAgeV1 and V2 versus others, not satisfying rule 1) and sometimes the same biomarker changing in opposite direction across studies (PCHorvath2, not satisfying rule 2). This example suggests that the interventional effects of senolytics, at least from an epigenetic aging perspective, may be inconsistent.

### Reliable generation 2+ biomarkers demonstrate the highest responsiveness

**Fig. 4:**
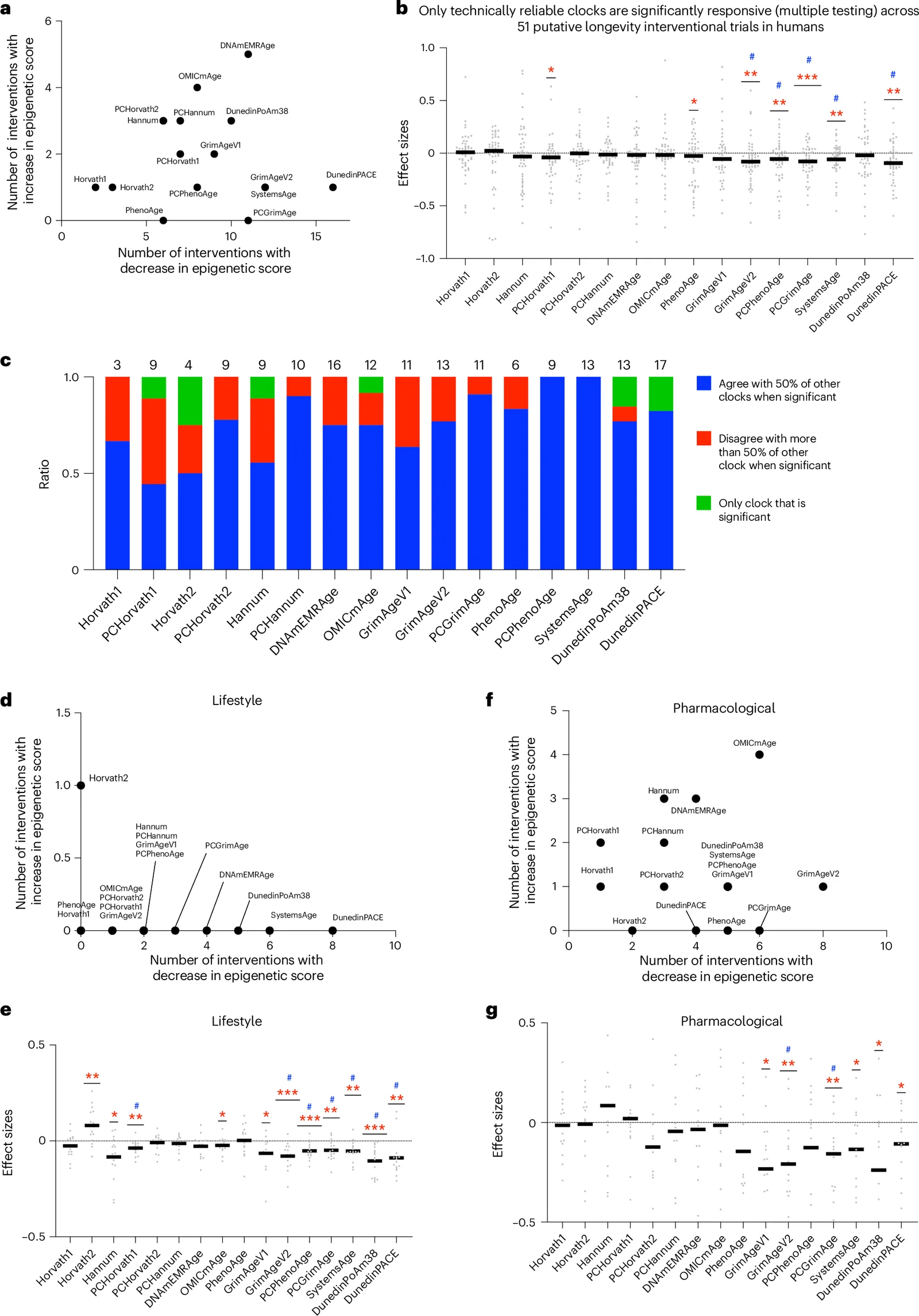
Response of 16 prominent DNAm biomarkers to interventions. **a**, A scatter plot showing the number of interventions for which each DNAm biomarker decreases (*x* axis) or increases (*y* axis). **b**, A scatter plot of effect sizes of interventions on DNAm biomarkers in which each point represents one intervention study: Significant effect sizes are marked with red asterisks and blue hashtags (*P < 0.05, **P < 0.01, ***P < 0.001, # significant after multiple-testing corrections), whereas solid lines indicate the mean effect size. **c**, A bar plot showing agreement and disagreement among DNAm biomarkers. For each clock, the number of interventions leading to significant changes were counted (denoted by the numbers at the top). Significant changes for one clock were then categorized as either being in agreement with >50% of other clocks (blue), being in disagreement with >50% (red) or being the only clock to show a significant change (green) **d**, A scatter plot of the number of lifestyle interventions for which each DNAm biomarker decreases (*x* axis) or increases (*y* axis). **e**, A categorical scatter plot of effect sizes of lifestyle interventions on DNAm biomarkers where each point represents one intervention study. Significant effect sizes are marked with red asterisks (*P < 0.05, **P < 0.01, ***P < 0.001, # significant after multiple-testing corrections); solid lines indicate the mean effect size. **f**, A scatter plot of the number of pharmacological interventions for which each DNAm biomarker decreases (*x* axis) or increases (*y* axis). **g**, A categorical scatter plot of effect sizes of pharmacological interventions on DNAm biomarkers where each point represents one intervention study. Significant effect sizes are marked with red asterisks (*P < 0.05, **P < 0.01, ***P < 0.001, # significant after multiple-testing corrections); solid lines indicate the mean effect size.

In addition to comparing interventions, we aimed to compare the responsiveness of the 16 DNAm biomarkers discussed previously. As an initial survey, we counted the number of interventions for which each DNAm biomarker decreased or increased significantly and compared these on a scatter plot (Fig. 4a and Supplementary Table 6). Most generation 2+ reliable biomarkers such as SystemsAge, GrimAgeV2, PCGrimAge, PCPhenoAge and DunedinPACE, decreased significantly in multiple interventions and increased in none or few interventions. Notably, the only generation 2+ biomarkers that increased in more than one study were GrimAgeV1 and DunedinPoAm38, which supports utilizing their more reliable counterparts instead. Meanwhile, most generation 1 biomarkers, such as Horvath1, Horvath2 and Hannum, sporadically increased or decreased in a handful of different interventions, lacking any clear trend. DNAmEMRAge and OMICmAge changed in many interventions, though interestingly, they were a mix of increases and decreases, which may reflect the complexity of the multi-omic and phenotypic data on which they were trained. Among all the biomarkers, DunedinPACE was the most responsive to a deflection in biological age, significantly decreasing in 16 interventions and increasing in only 1. To further quantify responsiveness, we performed a second analysis where we plotted all the effect sizes for a single clock from all interventions and performed a one-way *t*-test checking for deviations from zero for each clock. We reasoned that because all interventions in our dataset are targeted at ameliorating pathological aging potentially by reducing biological age, then if the DNAm biomarkers are indeed responsive they should show a significant decrease when all effect sizes were pooled together (Fig. 4b and Supplementary Table 7). This analysis revealed that all generation 2+ reliable biomarkers showed significant decreases. These include GrimAgeV2 (mean of −0.0814, *P* = 0.0075), PCPhenoAge (mean = -0.08876, *P* = 0.003), PCGrimAge (mean of −0.07843, *P* = 0.0003), SystemsAge (mean of −0.05859, *P* = 0.0043) and DunedinPACE which had the largest decrease (mean of −0.0891, *P* = 0.0012) (significant after multiple-testing correction). By contrast, only a single generation 1 (reliable) clock significantly decreased across all interventions (PCHorvath1 mean of −0.06674, *P* = 0.029), and a single generation 2 nonreliable DNAm biomarker decreased (PhenoAge mean of −0.06735, *P*= 0.0408) (not significant after multiple-testing correction). We also regressed out DNAm imputed cell fractions and observed that nearly all biomarkers that were responsive before cell fraction regression remained responsive even after accounting for cell fractions (Extended Data Fig. 1b). Overall, both analyses converged on the same insight: reliable second-generation biomarkers are responsive to interventions, with DunedinPACE showing the largest effect size and PCGrimAge exhibiting the strongest statistical significance. This suggests potentially two criteria for a biomarker to be responsive: (1) it needs to be reliable, and (2) it needs to be a mortality or rate of aging predictor.

### Reliable generation 2+ biomarkers align most with other biomarkers

When selecting epigenetic clocks to evaluate intervention responses, one may want to utilize a clock that is likely to agree with other clocks on the intervention effect. If the clock were to disagree with others, then the result would be confusing to interpret, or there would be a concern that the effect was a sporadic significant result (false positive). Thus we quantified the concordance of the 16 DNAm biomarkers—the likelihood that if a DNAm biomarker detected a significant effect then others would agree on the effect—analyzed across all interventions. Thus, for each intervention where at least one biomarker responded significantly, we compared each significant biomarker with the other significant biomarkers in that study. If the biomarker agreed with greater than 50% of the remaining biomarkers, we categorized this as an agreement, if less than 50%, then a disagreement, and if it was the only significant biomarker, then as a third category of being ‘only significant’. Finally, for each of the 16 biomarkers, we divided the number in each category by the total number of significant results for that clock. We observed two trends (Fig. 4c and Supplementary Table 8). First, generation 1 biomarkers had less agreement with other biomarkers (Horvath1 of 0.66, Horvath2 of 0.5 and Hannum of 0.55) as compared with their generation 2+ counterparts (DNAmEMRAge of 0.75, OMICmAge of 0.75, GrimAgeV1 of 0.63, PhenoAge of 0.83 and DunedinPoAm38 of 0.77). Second, reliable versions showed greater agreement compared with their original versions (Horvath2 0.5 versus PCHorvath2 0.77; Hannum 0.55 versus PCHannum 0.9; GrimAge 0.6 versus PCGrimAge 0.9; PhenoAge 0.83 versus PCPhenoAge 1; and DunedinPoAm38 0.76 versus DunedinPACE 0.82) with one exception (Horvath1 0.66 versus PCHorvath1 0.44). Of particular interest were cases where a given clock was the only one that showed any significant change. Most biomarkers that showed at least one result where they were the ‘only significant’ clock also had many other results where they had large disagreements with other clocks. We interpret these clocks as having a high likelihood of false positives. However, there is one notable exception: DunedinPACE, which was unique in that it never disagreed with other clocks, yet it was the only significant clock in 17.6% of the interventions where it was significant. Although it is still possible these ‘only significant’ clock results for DunedinPACE are false positives, it is also possible that DunedinPACE is simply the most sensitive clock to intervention effects or can detect unique intervention effects.

### Different DNAm biomarkers are sensitive to different intervention categories

We further wanted to test whether different DNAm biomarkers are responsive to different categories of interventions. To test this, we replicated our analysis from Fig. 4a,b, but this time limited to lifestyle and pharmacological interventions analyzed separately. In lifestyle interventions (Fig. 4d and Supplementary Table 9), DunedinPACE reported significant decreases in 8 out of the 15 interventions followed by SystemsAge (6), DunedinPoAm38 (5), DNAmEMRAge (4) and PCGrimAge (3). One-way *t*-tests revealed (Fig. 4e and Supplementary Table 10) that DunedinPoAm38 reported the largest decrease in effect size (mean of −0.1048, *P* = 0.0001), followed by DunedinPACE (−0.08887, 0.0037) and GrimAgeV2 (−0.07904, 0.0004) (all significant after multiple-testing corrections).

In comparison, after pharmacological interventions (Fig. 4f and Supplementary Table 11), GrimAgeV2 reported the highest number of significant decreases (8 out of 14) followed by PCGrimAge and OMICmAge (6). The top 4 biomarkers with largest decrease in effect size in the one-way *t*-test analysis (Fig. 4g and Supplementary Table 12) were DunedinPoAm38 (mean of −0.2394, *P* = 0.0442), GrimAgeV1 (−0.2332, 0.0151), GrimAgeV2 (−0.2092, 0.0058) and PCGrimAge (−0.1575, 0.0095) (only GrimAgeV2 and PCGrimAge significant after multiple-testing correction). Although several biomarkers did overlap between the top categories, many biomarkers were sensitive to the intervention type and their magnitude of effect size differed between the two interventional categories. Most notably, DunedinPACE was altered by fewer pharmacological interventions than other second-generation reliable biomarkers, despite its high responsiveness in lifestyle (Fig. 4d) and in uncategorized interventions (Fig. 4a). Meanwhile, GrimAgeV2 was the most sensitive to pharmacological interventions, but only changed in a single lifestyle intervention. Thus, different biomarkers were most sensitive to different intervention categories.

### Study population health status is critical to DNAm biomarker response

**Fig. 5:**
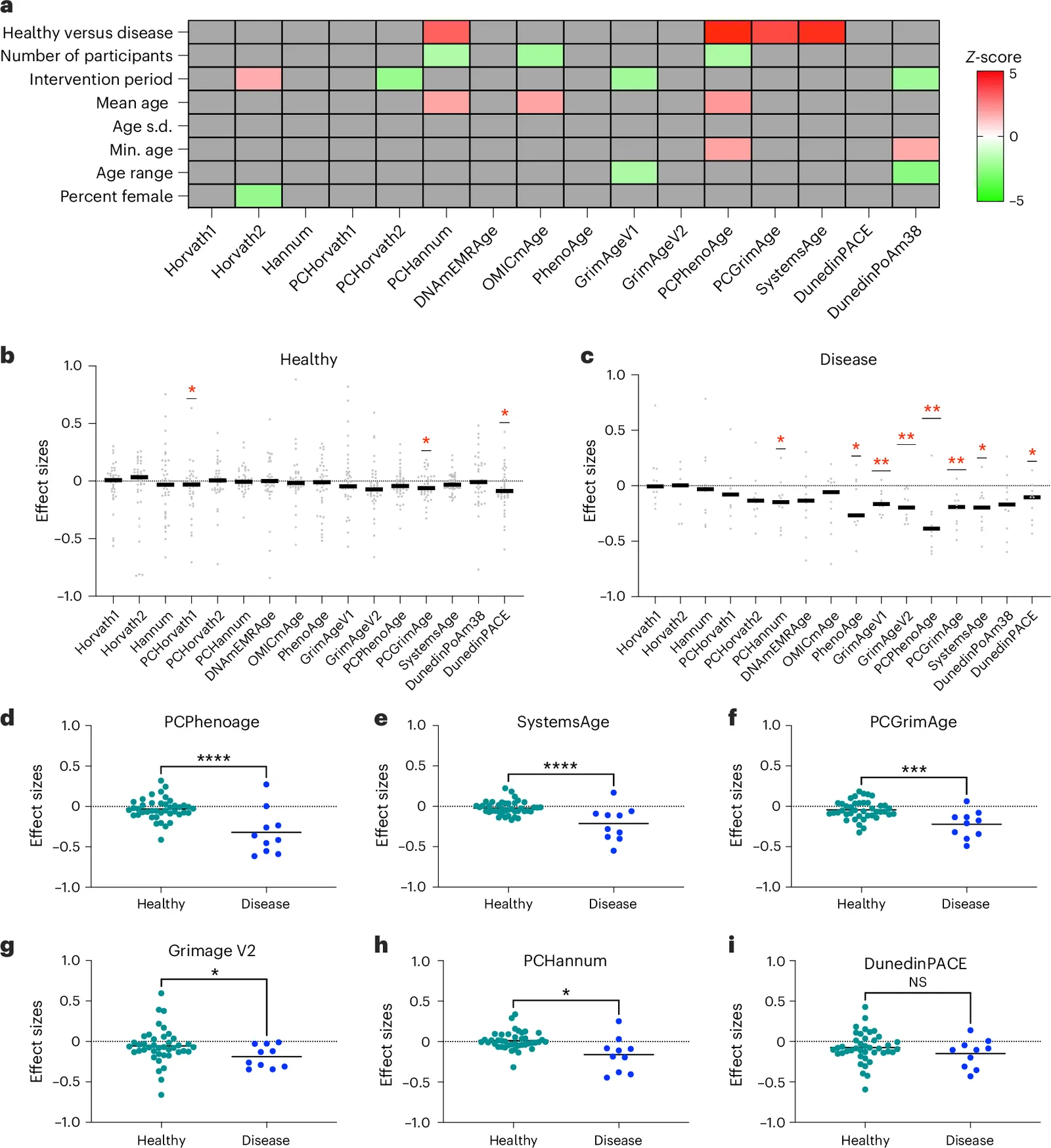
Effect of study characteristics on DNAm biomarker response to interventions. **a**, A heat map showing standardized association strengths (*Z*-scores) from a linear model relating study characteristics (across all interventions) to DNAm biomarker effect sizes. Red indicates that a study characteristic is associated with higher effect sizes, green indicates lower effect sizes and gray indicates no significant association. ‘Healthy’ refers to studies conducted in participants without a diagnosed disease condition and serves as the reference group. ‘Disease’ refers to studies involving participants with a specified clinical diagnosis or pathological condition. In this coding framework, the healthy group is the baseline; therefore, positive (red) values indicate that studies conducted in disease populations are associated with larger DNAm effect sizes compared with healthy populations. min. age, minimum participant age reported in the study; age s.d., standard deviation of participant age within the study. **b**,**c**, Categorical scatter plots illustrating DNAm biomarker effect sizes in studies conducted in healthy individuals (**b**) and individuals with disease (**c**). Significant effects are marked with red asterisks (*P < 0.05, **P < 0.01, ***P < 0.001, # indicates significance after multiple-testing correction). **d**–**i**, Categorical scatter plots comparing effect sizes between healthy and disease populations for specific DNAm biomarkers: PCPhenoAge (**d**), SystemsAge (**e**), PCGrimAge (**f**), GrimAgeV2 (**g**), PCHannum (**h**) and DunedinPACE (**i**). NS, not significant.

Given the heterogeneity across interventional studies, we examined whether clinical covariates influenced DNAm biomarker responsiveness. Using linear regression, we assessed associations between DNAm effect sizes and eight study-level variables: population health status (healthy versus disease), sample size, intervention duration, mean age, age s.d., minimum age, age range and percent female (Fig. 5a and Supplementary Table 13). Population health status showed the strongest association with highly responsive biomarkers (PCPhenoAge Z of 4.83; PCGrimAge of 4.07; SystemsAge of 4.65).

We then tested whether DNAm biomarkers responded differently in healthy versus disease populations. One-sample *t*-tests revealed that (Fig. 5b,c and Supplementary Tables 14 and 15) several biomarkers were significantly responsive in disease groups (for example, PCPhenoAge mean of −0.32, *P* = 0.0057; PCGrimAge of −0.22, *P* = 0.0022; SystemsAge of −0.213, *P* = 0.0105; all significant after multiple-testing correction), whereas only a few showed responsiveness in healthy groups (for example, DunedinPACE of −0.074, *P* = 0.0143; PCGrimAge of −0.043, *P* = 0.0191; none were significant after multiple-testing correction).

To determine whether this pattern reflected a few disease studies (*n* = 10) versus the larger number of healthy studies (*n* = 41), we compared biomarker effect sizes between groups using unpaired *t*-tests (Fig. 5d–i and Supplementary Table 16). Several biomarkers, including PCPhenoAge (mean difference of 0.28, *P* < 0.0001), SystemsAge (mean difference of 0.21, *P* < 0.0001) and GrimAgeV2 (mean difference of 0.14, *P* = 0.02), showed significantly larger decreases in disease populations. Even PCGrimAge, which was responsive in both groups, showed a greater response in disease studies (mean difference of 0.18, *P* = 0.002). Only DunedinPACE responded similarly in both populations (mean difference of 0.07, *P* = 0.25), suggesting it may be the most robust across varying health contexts.

### GenX DNAm biomarkers offer mechanistic insights into responsiveness

**Fig. 6:**
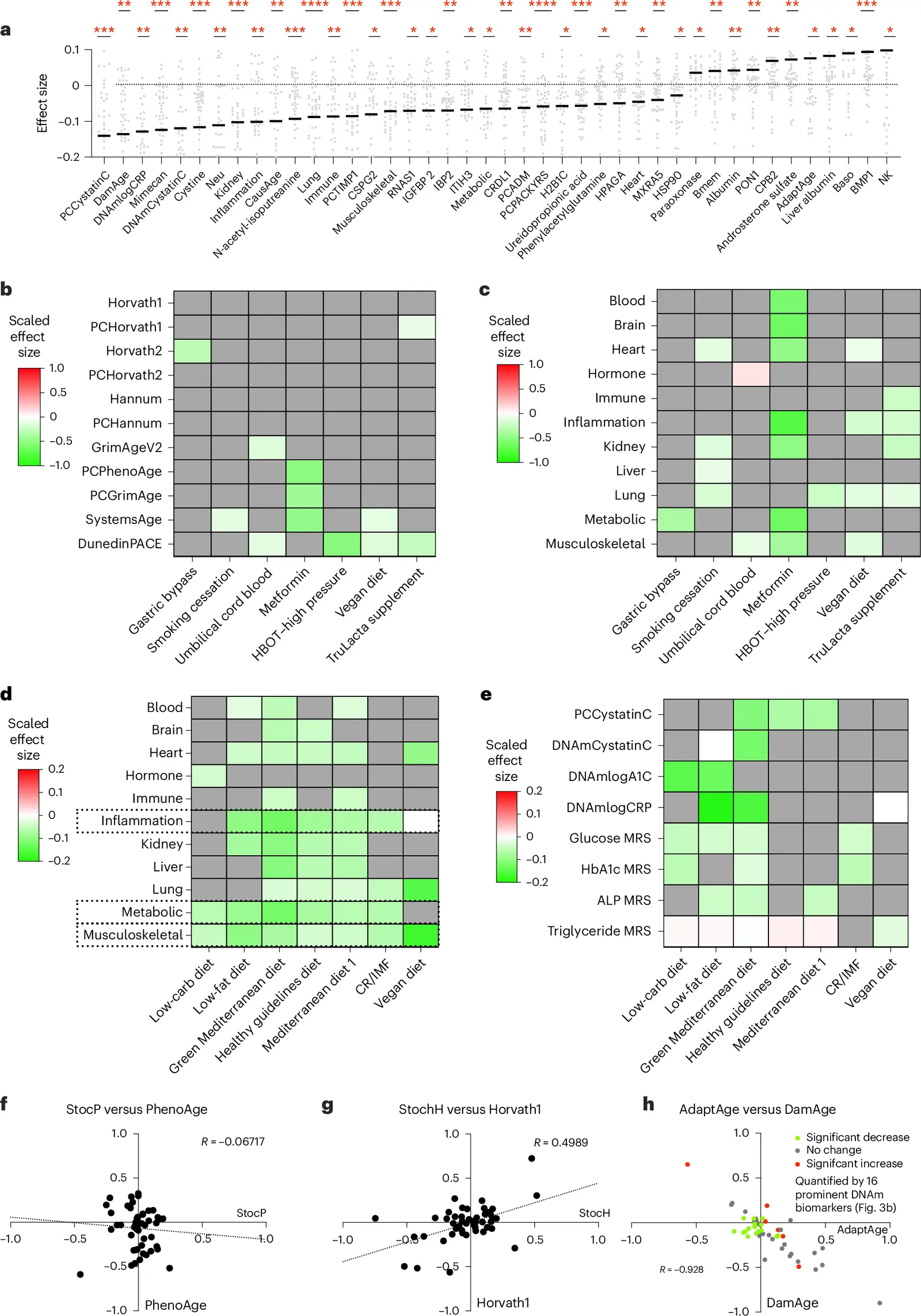
Explainable DNAm biomarkers (GenX) show specific changes and provide mechanistic insight. **a**, A scatter plot showing intervention effect sizes for GenX DNAm biomarkers. The *x* axis lists individual GenX biomarkers, and the *y* axis shows the corresponding standardized effect sizes. Each point represents the estimated effect size for a biomarker across interventions. Horizontal dashed lines indicate significance thresholds. Red points and red asterisks denote significant effects (*P < 0.05, **P < 0.01, ***P < 0.001), whereas gray points indicate nonsignificant effects. *P* values test whether the intervention-associated effect size differs significantly from zero. **b**, A heat map of whole-body aging DNAm biomarkers (*y* axis) across interventions (*x* axis). Red indicates an increase in epigenetic age, green indicates a decrease in epigenetic age and gray indicates no significant effect. **c**, A heat map of system-specific DNAm biomarkers (*y* axis) across interventions (*x* axis). The color scale is the same as in **b**. **d**, A heat map of scaled effect sizes for organ-system-specific DNAm biomarkers across dietary interventions. The color scale is consistent with **b** and **c**. **e**, A heat map of scaled effect sizes for DNAm-based metabolite and protein proxies across dietary interventions. The color scale is consistent with **b**–**d**. **f**, A scatter plot comparing intervention effect sizes estimated by StocP (*x* axis) and PhenoAge (*y* axis). Each point represents a single intervention. Red points indicate interventions associated with significant increases in epigenetic age across the 16 aging biomarkers, green points indicate interventions associated with significant decreases in epigenetic age and gray points indicate interventions with no significant overall effect. **g**, A scatter plot comparing intervention effect sizes estimated by StocH (*x* axis) and Horvath1 (*y* axis). Each point represents a single intervention and is colored according to its overall effect on epigenetic age as described for **f**. **h**, A scatter plot comparing intervention effect sizes estimated by AdaptAge (*x* axis) and DamAge (*y* axis). Each point represents a single intervention and is colored according to its overall effect on epigenetic age as described for **f**.

Clocks such as SystemsAge, GrimAge and OMICmAge were built from component DNAm biomarkers to better explain the specific aging changes that they are capturing. Thus, we also tested responsiveness for 78 generation explainable (GenX) DNAm biomarkers (Supplementary Table 17). We observed 39 biomarkers with significant changes across all interventions (Fig. 6a). Examples of biomarkers with particularly high responsiveness included PCGrimAge components PCPACKYRS (effect size of −0.05824, *t* = 4.636), PCCystatinC (−0.1404, 3.630) and PCTIMP1 (−0.08526, 3.666); SystemsAge components kidney (−0.1021, 3.753), lung (−0.08812, 4.615) and musculoskeletal (−0.07132, 3.729); and finally, OMICmAge components EBP-*N*-acetyl-isoputreanine (−0.09316, 3.506), EBP-ureidopropionic acid (−0.05658, 3.733), EBP-Mimecan (−0.1238, 4.047), EBP-Cystine (−0.1162, 3.897), EBP-CBPB2 (0.06373, 3.497) and EBP-BMP1 (0.09398, 4.183).

**Extended Data Fig. 2:**
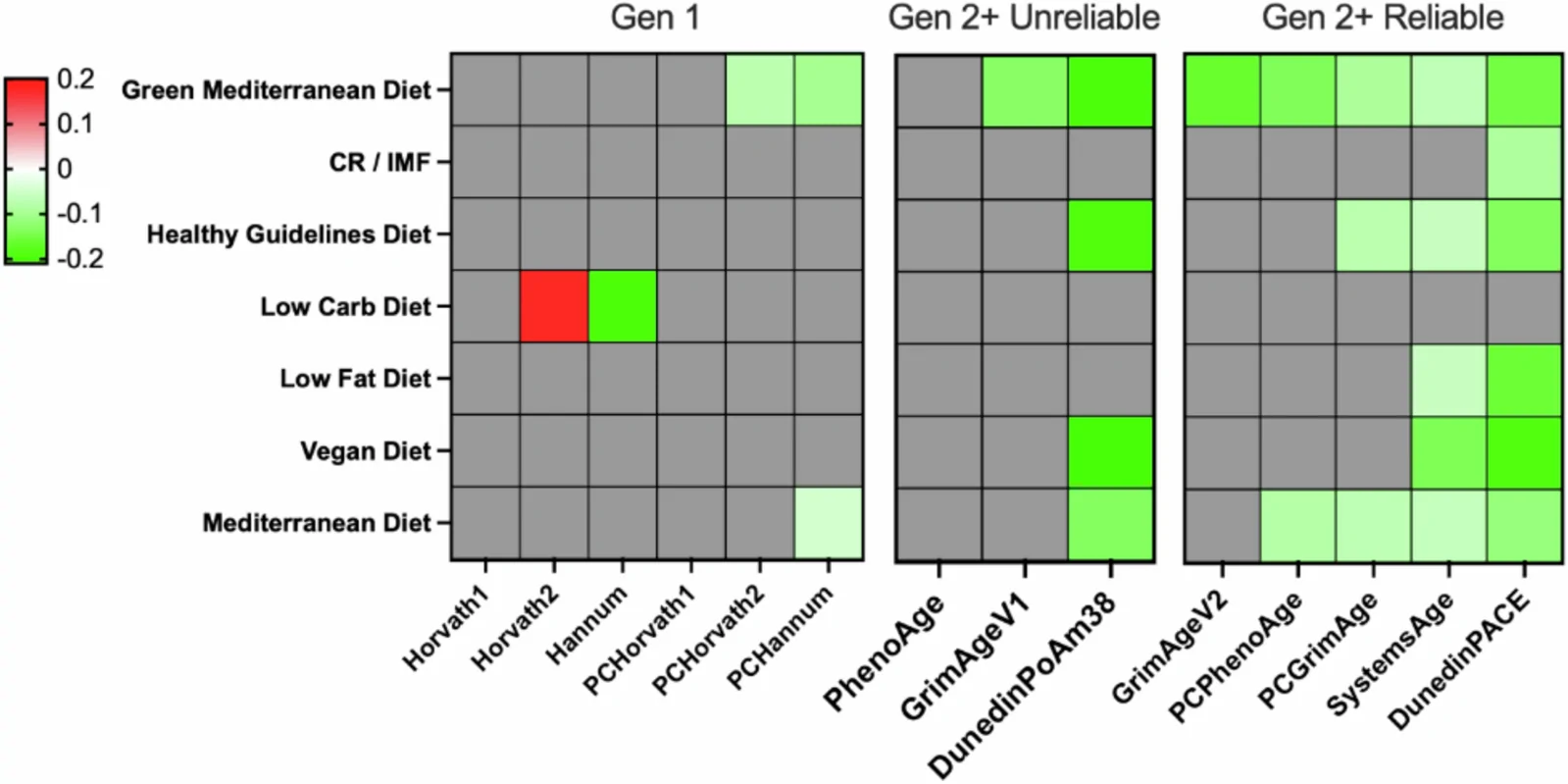
Dietary interventions demonstrate differential responsiveness across generations of DNA methylation biomarkers. Heatmaps summarizing standardized effect sizes of dietary interventions across first-generation, second-generation, and reliable DNA methylation biomarkers, illustrating that reliable second-generation biomarkers exhibit the most consistent reductions in biological age.

To understand how GenX clocks can provide more insight into responsiveness, we examined specific intervention, selected on the basis of whether they fell into two categories: (1) significantly decreasing epigenetic age in only a handful of generation 2 reliable clocks (smoking cessation—SystemsAge; umbilical cord blood transfusion—DunedinPACE and GrimAgeV2; metformin—SystemsAge, PCGrimAge and PCPhenoAge; hyperbaric oxygen therapy—DunedinPACE; Vegan Diet—DunedinPACE and SystemsAge; TruLacta supplement—DunedinPACE) or (2) not significantly decreasing epigenetic age in any generation 2 reliable clocks (gastric bypass) (Fig. 6b and Supplementary Table 18). The sporadic responsiveness of clocks to the first category of interventions may suggest they are false positives, whereas the lack of responsiveness in the second category may suggest they do not modify epigenetic age. However, we considered the possibility that these interventions simply had very specific effects that may not be detectable using a general aging clock. Accordingly, the SystemsAge components suggested these interventions may be impacting different systems (Fig. 6c, Extended Data Fig. 2 and Supplementary Table 18). For example, the two interventions that did not modify any general Gen 2 reliable clock do show significant decreases in epigenetic age of specific systems (gastric bypass decreases metabolic score with effect size 0.43). Other interventions include smoking cessation seeing maximal decrease in lung (0.20), umbilical cord transfusion in musculoskeletal (0.13) and hyperbaric oxygen therapy in lung (0.27). Although other systems saw multi-system decreases such as metformin with inflammation (0.80), brain (0.70) and metabolic (0.70) decreasing the most. In the vegan diet, the largest decrease was seen in inflammation (0.23) and musculoskeletal (0.17). However, in TruLacta supplement, kidney (0.29), immune (0.28) and inflammation (0.24) reported the greatest impact after the intervention.

Finally, given we had a multitude of diets (seven) in our study (low-carb diet, low-fat diet, green mediterranean diet, healthy guidelines diet, red meat mediterranean diet, CR/IMF (calorie restriction/intermittent fasting) and vegan diet) we wished to understand if GenX DNAm biomarkers could provide deeper mechanistic understanding specifically into dietary interventions. We looked at the 11 system scores and specific metabolic related protein and metabolite epigenetic proxy biomarkers (Fig. 6d,e and Supplementary Table 18). Among the 11 system-specific biomarkers, musculoskeletal was the only score that significantly decreased across all seven diets. Meanwhile, two system scores (metabolic and inflammation) were responsive in six of the seven diets. When looking at the protein and metabolite proxies, we observed the epigenetic proxy of triglycerides to be significant across six of the seven diets, whereas the glucose proxy was significant in four of seven diets, suggesting great potential for these epigenetic proxies in measuring diet-based interventions.

### Responsiveness of stochastic and causal components

Recent advances have led to a new generation of DNAm biomarkers that aim to separate meaningful biological signals from random noise^19,20^. These biomarkers appear to contain both stochastic (random) and nonstochastic (predictable, biologically driven) components. These studies found that first-generation clocks such as Horvath1, which estimate chronological age, mostly reflect stochastic epigenetic noise. By contrast, second-generation clocks (generation 2+) such as PhenoAge and GrimAge are believed to capture more biologically meaningful, nonrandom aging signals. However, those findings were based on cross-sectional data. To test this more rigorously, we used the longitudinal datasets in TranslAGE^18^.

We examined how much of the change in each clock could be attributed to stochastic versus nonstochastic components. For example, we found that Horvath1 was strongly correlated with its stochastic component (*R* = 0.50), whereas PhenoAge showed almost no correlation with its own stochastic component (*R* = −0.06), suggesting that PhenoAge reflects more structured biological aging (Fig. 6f,g).

Clocks have also been developed to isolate causal DNAm changes that may reflect either harmful or adaptive aging processes^16^. We revisited the DamAge–AdaptAge framework and found a strong inverse relationship between their changes (*R* = −0.89). Yet, most interventions reduced both components, indicating an overall slowdown of biological change rather than a shift from harmful to adaptive processes.

## Discussion

Epigenetic clocks, falling under the umbrella of DNAm biomarkers, have long been proposed as surrogate endpoints for longevity clinical trials^2,21^. Although their prognostic value (that is, association with morbidity and mortality) is well established^22–24^, their responsiveness to interventions has not been systematically tested across multiple trials and biomarkers^25^. To address this, we assembled TranslAGE^18^, a curated database of 51 longitudinal intervention studies with pre–post blood-based methylation data (total of 3,128 samples). We systematically calculated DNAm biomarkers and analyzed intervention effects in a harmonized pipeline.

Our results show that pharmacological interventions, particularly anti-TNF therapies^26,27^ and metformin^28–30^, consistently induced strong changes in DNAm biomarkers, more so than supplements, diets or lifestyle changes. These findings suggest that pharmacological agents may exert robust effects on aging biology, probably by targeting inflammation and metabolic pathways such as TNF, AMPK or mTOR.

We also evaluated the replicability of intervention effects and found that interventions modifying the same biomarkers consistently across studies, such as anti-TNF agents and the Mediterranean diet, had more reliable outcomes. This highlights the importance of methodological consistency and reproducibility when assessing biomarker responses in clinical trials. Although it is still unclear whether short-term biomarker changes correspond to long-term improvements in disease, healthspan or lifespan, it is encouraging that the most robust results concerning anti-TNF agents and the Mediterranean diet^31,32^ are supported by ample evidence from other lines of research. For example, prior studies have shown that anti-TNF agents can extend lifespan in mice^33^. These converging findings suggest that the corresponding biomarkers should continue to be tested as possible surrogate endpoints in the future, even as longer-term validation remains essential. It should be noted that pharmacological interventions tend to produce larger average effect sizes on DNAm biomarkers, whereas dietary interventions show greater cross-biomarker consistency, emphasizing that effect magnitude and consistency capture distinct and complementary dimensions of biomarker response.

Importantly, we found that second-generation (generation 2+) DNAm biomarkers, such as PCGrimAge^12^, SystemsAge^11^ and DunedinPACE^10^, showed the strongest and most consistent responsiveness. Among these, DunedinPACE showed the largest effect sizes, whereas PCGrimAge yielded the most statistically significant results. These clocks were robust across intervention types and populations, underscoring their value in both research and clinical trial settings. This suggests that future clinical trials assessing epigenetic clocks should focus on these clocks rather than chronological age clocks or low-reliability clocks.

We observed that the health status of the study population influenced biomarker responsiveness^34^. DNAm biomarkers were more responsive in disease populations compared with healthy individuals. For example, PCPhenoAge and SystemsAge showed greater reductions in disease populations, probably reflecting higher baseline biological dysregulation. This pattern is consistent with prior geroscience findings that interventions often have stronger observable effects in populations with greater physiological impairment, where there is more room for improvement^35,36^. One explanation relates to the training populations used to develop specific biomarkers. Biomarkers trained in older or more clinically diverse cohorts are more sensitive to changes in dysregulated systems, which helps explain why measures such as PCPhenoAge and SystemsAge are particularly responsive in disease populations. Conversely, DunedinPACE, which was trained in younger and healthier individuals, has shown greater sensitivity in healthier populations. Together, these findings suggest that stratifying participants by health status, while considering the training context of the biomarker, may optimize detection of intervention effects and improve trial design.

We further demonstrated that GenX ‘explainable’ DNAm biomarkers, those targeting specific physiological systems, can provide mechanistic insights into which organ systems are affected. For example, smoking cessation selectively reduced lung system scores, whereas metformin influenced inflammatory, brain and metabolic pathways. These system-level scores offer a complementary approach to global clocks and may guide the development of targeted aging therapies. In our analysis, we also observed several principal component (PC) biomarkers (for example PCCystatinC, PCADM and PCPACKYRS) showed stronger and more reproducible responsiveness to interventions than many composite clocks. As these biomarkers are pathway proximal, they often capture earlier and larger shifts in inflammation, cardio–renal function and metabolic signaling^37–40^. For longevity trials, incorporating a focused panel of PC biomarkers may provide higher sensitivity to change and greater mechanistic interpretability than integrative measures alone^41^. An additional consideration is that integrative biomarkers may ‘average out’ effects across components. For example, if one PC biomarker increases while another decreases, the composite measure may show little net change. Similarly, if only a single PC biomarker shifts, this effect may be diluted when combined with others. This averaging could explain why certain composite clocks appear less responsive in our analysis.

Finally, we examined stochastic and causal components of DNAm changes. Our data support prior claims that generation 1 clocks such as Horvath1 largely reflect stochastic epigenetic noise, whereas generation 2+ biomarkers capture more structured, biologically meaningful change^19^. Importantly, our analysis indicates that intervention effects are more likely to register in the structured, nonstochastic components rather than the stochastic background. This helps clarify a longstanding debate: although stochasticity dominates early clocks, these noisy signals are largely insensitive to interventions. By contrast, biomarkers that track pathway-proximal, causal components, particularly those anchored in inflammation, metabolic and cardio–renal axes, show consistent responsiveness to treatment. This suggests that the ability of a biomarker to detect intervention effects depends on the extent to which it captures nonstochastic, biologically directed variation rather than random epigenetic drift. We also validated prior findings that effective interventions reduce damaging causal signals (DamAge) and, in some cases, increase adaptive ones (AdaptAge). Notably, DamAge and AdaptAge appeared to register effects from a distinct set of interventions compared with the other clocks analyzed, which should be further investigated. This illustrates not all biomarkers are interchangeable in intervention studies and that deploying a portfolio of measures may provide a more complete picture of how different classes of interventions act on the aging process^16,20^.

Limitations of this study should be acknowledged. First, we pooled effect sizes across heterogeneous interventional studies that differed substantially in participant age ranges, intervention modalities, durations and study designs. Although this heterogeneity limits biological specificity and precludes interpretation of pooled effects as mechanistic equivalence, our intent was descriptive rather than causal: to characterize broad patterns of DNAm biomarker responsiveness and prioritize clocks that show consistent sensitivity to change. Accordingly, biological interpretation is emphasized within intervention classes, which are analyzed separately throughout the Analysis. Second, variability in study quality, sample size and intervention relevance is an inherent limitation; however, this diversity provides a wide distribution of effect sizes that is informative for benchmarking biomarker behavior rather than evaluating individual intervention efficacy. Third, lack of harmonized preprocessing and batch correction across studies may affect comparability, and future updates to TranslAGE^42^ will incorporate such adjustments where metadata permit. Fourth, the interpretation of generation 2+ clocks requires caution, as their training on composite risk factors (for example, C-reactive protein in GrimAgeV2) means observed responsiveness may reflect the modulation of intermediate health or inflammatory states rather than direct effects on aging biology per se. Fifth, the absence of an empirically defined minimal clinically important difference for DNAm clocks remains a key barrier to their use as surrogate endpoints; future work must anchor clock changes to clinically meaningful functional, physiological or disease-specific outcomes. Finally, although standardized effect sizes may appear modest, for widely used clocks such as Horvath, Hannum, PhenoAge and GrimAge, where the standard deviation of epigenetic age deviation is typically on the order of ~4–6 years, an effect size of ~0.2 corresponds to an approximate 1-year shift in epigenetic age, indicating that the observed changes are not biologically trivial.

This study provides a large-scale, systematic evaluation of DNAm biomarker responsiveness across longevity interventions. Our findings support the prioritization of generation 2+ clocks, particularly DunedinPACE and PCGrimAge, in clinical trials. They also highlight the utility of system-specific biomarkers and the importance of population health stratification. We encourage future work to move beyond prognostic validation and rigorously test responsiveness and surrogate endpoint potential. Doing so will accelerate the clinical translation of DNAm biomarkers and help bring aging interventions into mainstream medical practice.

## Methods

### Analysis pipeline

#### Step 1: data curation

We began by compiling a comprehensive list of publicly and privately available clinical trials and studies focused on longevity interventions. Publicly available datasets were sourced from the GEO and EMBL repositories, whereas private datasets were obtained from TruDiagnostic. Datasets were selected on the basis of the following criterion: (1) interventional study or trial is of a intervention that has been hypothesized for the purposes of longevity or healthspan extension as defined in literature, (2) DNAm data is available pre and post intervention, (3) the tissue of origin for the DNAm data is blood (ideally peripheral blood mononuclear cells), (4) study passes quality checks such as: minimum number of individuals in the treatment arm and consistent methodology of DNAm data generation at pre and post time points. For each study, we curated extensive study-level metadata, including the following key variables:

- Intervention type: identifying whether the intervention involved lifestyle changes, pharmacological treatments or other longevity-enhancing approaches
- Diseases in population: listing any comorbidities or conditions present in the study population that could influence the effects of the intervention
- Age range and percent female: capturing demographic distribution to understand age- and sex-related response variability

These metadata were manually extracted from study documentation and supplementary materials to ensure accuracy.

#### Step 2: metadata standardization

We utilized a standardized cleaning script that was adapted for each dataset. We standardized the variables across all studies to create uniform, comparable datasets. This harmonization process involved:

- Standardized columns: We ensured that all datasets contained consistent variables, including age, sex, sample ID, individual ID, follow-up time from baseline draw and sample type (control versus participant). This step enabled consistent cross-study analysis.
- Sample identification: to maintain data integrity, each sample was assigned a unique sample ID and linked to an individual ID, ensuring that repeated measurements from the same individual could be tracked longitudinally.

This harmonization facilitated subsequent analyses, facilitating robust comparisons between different studies and intervention types.

#### Step 3: clock calculation

For each of the curated datasets, we calculated more than 110 DNAm biomarkers using the MethylCIPHER 2.0 package (updated to include more clocks). This package enables the computation of various methylation-based aging and health biomarkers, such as

- Epigenetic clocks (for example, Horvath, Hannum clock)
- Health-related markers (for example, DNAmPackYears)
- Protein and metabolite proxies (for example, DNAmGDF15 and MRS triglyceride)

These biomarkers were calculated for each sample on the basis of the methylation data available in each study. A list of all the biomarkers calculated with additional information on them can be found in Supplementary Table 1.

#### Step 4: age-residualized scores

We processed the biomarkers by age-residualizing them in each dataset. Age-residualization was performed to control for the natural variation in DNAm biomarkers due to chronological age. The procedure involved linear regression. We used age as the independent variable and each DNAm biomarker as the dependent variable to calculate the residuals. These age residuals represent the portion of the biomarker that is not explained by chronological age, allowing us to isolate the effect of the intervention on DNAm biomarkers.

#### Step 5: biomarker standardization

To ensure comparability across datasets, we standardized the calculated biomarkers. To do this, we calculated the standard deviation in the Health and Retirement Study (EPIC array, whole blood) for all age-residualized DNAm biomarkers^43^. After linear regression, the means of the age residuals for all biomarkers were already zero. We thus divided the age residuals for each DNAm biomarker in all datasets by the standard deviation for that biomarker calculated in the reference datasets, which puts them all on the same scale and thus comparable to each other.

#### Step 6: paired *t*-test

As all our studies had both pre- and postintervention samples, we performed paired *t*-tests to evaluate the effect of the intervention on the calculated DNAm biomarkers. This analysis was conducted as follows:

- Pre- versus postintervention comparison: for each individual, the change in age-residualized biomarker values between pre- and postintervention samples was calculated. The paired *t*-test was used to assess whether these changes were statistically significant.
- Significance threshold: a *P* value of <0.05 was considered statistically significant.

#### Step 7: heat map and follow-up analysis

To further explore the responsiveness of DNAm biomarkers to longevity interventions, we generated heat maps and performed additional statistical analyses:

- One-way *t*-tests: conducted to test for deviations from zero effect size in intervention responses
- Exploratory analyses: multiple analyses, including correlation tests and regression models, were used to explore relationships between biomarker changes and other variables such as age, sex and intervention type

### Ethics statement

This study is a secondary analysis of previously published human intervention studies and publicly available or collaborator-provided datasets. No new human participants were recruited for the present secondary analysis. The secondary data analysis protocol was approved by the Yale Institutional Review Board (IRB) (study number 2000040453). All original studies received approval from their respective IRBs or ethics committees, and all participants provided written informed consent. For the previously published studies, original IRB approval and consent information can be found in the original papers (Supplementary Table 1). The metformin study was approved by the Committee on Human Subjects of the University of Hawaii, and written informed consents were obtained from all participants, including a separate informed consent document agreeing to the use of their biobanked specimens for future research. For the AC11 supplement study, ethical approval was obtained from the Institute of Regenerative and Cellular Medicine (IRCM; approval number IRCM-2021-287, study number 01-AC11-001v3, approved 31 May 2021). The study was conducted under the oversight of principal investigator Phillip Gallegos, MD (Thrive Medicine Clinic), was sponsored by Optigenex and is registered at ClinicalTrials.gov (NCT05310123). All participants provided written informed consent before enrollment. For the hyperbaric oxygen therapy study, ethical approval was obtained from IRCM (approval number IRCM-2021-310, approved 8 December 2021; protocol number SM-TD-001). The study was conducted under the oversight of principal investigator Jason Sonners (New Jersey HBOT). All participants provided written informed consent before enrollment. For the botanical supplement (copaiba essential oil) study, the study was conducted by SF Research Institute (protocol DI202308081, principal investigator John Ademola, PhD, SF Research Institute), sponsored by dōTERRA International. The study was conducted in accordance with ICH E6 Good Clinical Practice guidelines and all applicable provisions of 21 CFR 812, and all participants provided written informed consent. The epigenetic analysis of deidentified samples was subsequently performed by TruDiagnostic under IRCM-approved retrospective protocol Tru-014-2025 (IRCM-2025-433, approved 26 March 2025; principal investigator Varun Dwaraka, PhD, TruDiagnostic). For the follistatin gene therapy study, ethical approval was obtained from the IRB of the Global Alliance for Regenerative Medicine (GARM Clinic, Próspera ZEDE, Roatán, Honduras). The study is registered at ClinicalTrials.gov (NCT06411366) and was sponsored by Minicircle. All participants provided written informed consent before enrollment. For the semaglutide study, participants were clients of Yunique Medical (principal investigator Lawrence Siegel, APRN) who registered biological samples through TruDiagnostic’s clinical testing platform and provided informed consent for deidentified data use in research at the time of sample registration, consistent with TruDiagnostic’s standard research consent framework. The retrospective epigenetic analysis was conducted under IRCM-approved protocol Tru-014-2025 (IRCM-2025-433, approved 26 March 2025; principal investigator Varun Dwaraka, PhD, TruDiagnostic).

### One-way *t*-tests for deviations from 0 effect size

To evaluate whether the observed effect size significantly deviated from zero, a one-sample *t*-test was performed using GraphPad Prism (version 9.4.0). The dataset included different measurements based on the condition being tested (for example: 51 for all interventions, 41 for healthy, 10 for disease). Test setup: in Prism, a one-sample *t*-test was conducted by specifying a hypothetical mean (Mu) of 0, which represents the expected effect size under the null hypothesis of no effect. The normality of the data was assumed, and Prism automatically performed the test using the standard *t*-test formula. Prism provided the *t*-statistic, degrees of freedom and two-tailed *P* value to evaluate whether the mean effect size significantly differed from zero. A significance level of 0.05 was used. All analyses and visualizations were conducted within Prism.

### Clinical trial covariates association analysis

To assess the association between DNAm biomarkers and clinical covariates, we first curated a comprehensive set of clinical variables from the study datasets. Clinical covariates were selected on the basis of their relevance to clinical trials. The covariates were extracted from study metadata or clinical records provided within each dataset. Where applicable, continuous variables were normalized or transformed to achieve better model fit. The association between DNAm biomarker effect sizes and clinical trial covariates was assessed using univariate regression models. This analysis aimed to identify covariates that significantly influenced DNAm biomarker responsiveness. For each DNAm biomarker, univariate linear regression models were fit to evaluate the independent association with each clinical covariate: biomarker = β0 + β1 × Covariate + ϵ. Covariates were tested individually for each biomarker, and the strength of association was assessed using *Z*-score, and absolute *Z*-scores less than 1.96 were considered not significant.

### Multiple-testing correction

#### Multiple-testing correction: independent testing via PCA

We used the independent testing framework proposed by Li et al. (2011)^44^, which replaces the raw number of tests with an effective number of independent tests derived from the correlation structure among variables.

##### Problem with standard corrections

Classical approaches such as Bonferroni assume that all hypothesis tests are independent. This assumption does not hold here, because both DNAm clocks and intervention effects are highly correlated. Clocks often rely on overlapping CpG sites and capture similar biological signals, whereas interventions of similar categories (for example, diets, exercise regimens and pharmacological agents) frequently elicit correlated physiological responses. Applying standard multiple-testing corrections in this context would be overly conservative, eliminating true biological signals.

##### PCA-based estimation of independent tests

Li et al^44^. proposed estimating the effective number of independent tests by applying PC analysis (PCA) to the correlation matrix of the tested variables. In our context, this involves:

- Computing correlation matrices across both clocks and interventions
- Performing PCA on each matrix
- Counting the number of independent PCs required to explain 90% of the total variance
- Defining this number of PCs as *M*ₑ, the effective number of independent tests

By using *M*ₑ rather than the raw number of clocks or interventions, the correction accounts for redundancy and avoids overpenalization.

##### Pragmatic justification in our setting

Given the high degree of intercorrelation among both clocks and interventions, the effective number of independent tests (*M*ₑ) is expected to be much smaller than the total number of comparisons. Thus, applying a nominal *P* < 0.05 threshold is effectively similar to using a Bonferroni-style correction based on *M*ₑ rather than the raw count of tests. In other words, our choice does not ignore multiple testing, but instead reflects the reduced dimensionality of the data.

##### Alignment with study goals

Our primary aim is to identify clocks and interventions that consistently show meaningful associations, not to rigidly test each pairwise comparison in isolation. The PCA-based independent testing framework respects the correlation structure in both sets of variables while preserving sensitivity to true positives. Moving forward, reporting the estimated *M*ₑ values will provide a transparent quantification of how much redundancy reduction is achieved in both clocks and interventions.

We have reported the effective number of independent tests we found for each analysis and in supplementary Table 20 and its corresponding Bonferroni *P* value cutoff.

### Reporting summary

Further information on research design is available in the Nature Portfolio Reporting Summary linked to this article.

## Online content

Any methods, additional references, Nature Portfolio reporting summaries, source data, extended data, supplementary information, acknowledgements, peer review information; details of author contributions and competing interests; and statements of data and code availability are available at https://doi.org/10.1038/s41591-026-04562-9.

## Supplementary information

Reporting summary

Peer review file

Supplementary Tables Glossary of Supplementary Tables.

Supplementary Table 1 TranslAGE datasets and biomarker catalog.

Supplementary Table 2 Significant biomarker counts by intervention category.

Supplementary Table 3 Mean effect sizes by intervention category.

Supplementary Table 4 Individual intervention × biomarker responses.

Supplementary Table 5 Overall intervention significance.

Supplementary Table 6 Biomarker responsiveness counts.

Supplementary Table 7 Overall responsiveness of each biomarker.

Supplementary Table 8 Biomarker concordance analysis.

Supplementary Table 9 Lifestyle intervention responsiveness counts.

Supplementary Table 10 Lifestyle intervention effect size analysis.

Supplementary Table 11 Pharmacological intervention responsiveness counts.

Supplementary Table 12 Pharmacological intervention effect size analysis.

Supplementary Table 13 Clinical trial covariate associations.

Supplementary Table 14 Healthy population analyses.

Supplementary Table 15 Disease population analyses.

Supplementary Table 16 Healthy versus disease comparisons.

Supplementary Table 17 Generation X biomarker analyses.

Supplementary Table 18 Mechanistic analyses of explainable biomarkers.

Supplementary Table 19 Stochastic and causal biomarker analyses.

Supplementary Table 20 Multiple testing correction framework.

## Data Availability

TranslAGE (https://www.translage.io) is being developed as an open resource for the geroscience community^18^. Accession numbers or controlled-access pathways for all datasets used in this study are listed in Supplementary Table 1 and on translage.io. (1) For datasets indicated as publicly, (indicated as ‘Public’ in Supplementary Table 1), harmonized datasets can be directly downloaded from the TranslAGE website; please refer to this video for further details on how to access data easily via TranslAGE. (2) The controlled-access dataset provided by Michael Corley (indicated as ‘Controlled access’ in Supplementary Table 1) can be applied for by emailing mjcorley@health.ucsd.edu. Access to participant-level data is controlled in accordance with privacy and ethical protections. Access will require IRB review, a data use agreement and approval from the clinical trial team. All access requests will be replied to within 2 months. Aggregate data are publicly available on the TranslAGE website and supplementary tables. (3) For controlled-access datasets provided by TruDiagnostic (indicated as ‘Partner datasets’ in Supplementary Table 1), access can be applied for via TruDiagnostic at https://www.trudiagnostic.com/softwarerequest/. Access requires approvals from both TruDiagnostic and the original research partner for each dataset; datasets will be made available to any academic researcher agreeing to a standard data use agreement confirming that the intended use is strictly for academic, nonprofit research. All access requests will be replied to within 2 months.

## Code availability

Code to calculate all clocks (except for OMICmAge and DNAmEMRAge) are available via GitHub at https://github.com/HigginsChenLab/methylCIPHER. Code to calculate OMICmAge, DNAmEMRAge and the associated underlying algorithm can be requested at https://www.trudiagnostic.com/softwarerequest/, for research purposes at no cost. Access is contingent upon the requestor agreeing to a standard, noncommercial software license agreement that confirms the intended use is strictly for academic, nonprofit research and that the requestor will not attempt to reverse engineer or commercially exploit the software and will be provided within 2 months. All R codes used to develop and validate EMRAge, DNAmEMRAge and OMICmAge are available via GitHub at https://github.com/LaskySuLab/OMICmAge/.
