# Supplementary material for: Responsiveness of epigenetic aging biomarkers to longevity interventions in humans
Source: Nat Med. 2026 Aug 21;32(9):3477–90. doi: 10.1038/s41591-026-04562-9 (PMC13577923; doi:10.1038/s41591-026-04562-9)
Supplement: Supplementary file 1 [file 41591_2026_4562_MOESM1_ESM.pdf]

nature portfolio

Corresponding author(s): Raghav Sehgal

Last updated by author(s): Jun 25, 2026

Reporting Summary

Nature Portfolio wishes to improve the reproducibility of the work that we publish. This form provides structure for consistency and transparency in reporting. For further information on Nature Portfolio policies, see our Editorial Policies and the Editorial Policy Checklist.

Statistics

For all statistical analyses, confirm that the following items are present in the figure legend, table legend, main text, or Methods section.

n/a	Confirmed	
☐	☒	The exact sample size (*n*) for each experimental group/condition, given as a discrete number and unit of measurement
☐	☒	A statement on whether measurements were taken from distinct samples or whether the same sample was measured repeatedly
☐	☒	The statistical test(s) used AND whether they are one- or two-sided<br>*Only common tests should be described solely by name; describe more complex techniques in the Methods section.*
☐	☒	A description of all covariates tested
☐	☒	A description of any assumptions or corrections, such as tests of normality and adjustment for multiple comparisons
☐	☒	A full description of the statistical parameters including central tendency (e.g. means) or other basic estimates (e.g. regression coefficient) AND variation (e.g. standard deviation) or associated estimates of uncertainty (e.g. confidence intervals)
☐	☒	For null hypothesis testing, the test statistic (e.g. *F*, *t*, *r*) with confidence intervals, effect sizes, degrees of freedom and *P* value noted<br>*Give P values as exact values whenever suitable.*
☒	☐	For Bayesian analysis, information on the choice of priors and Markov chain Monte Carlo settings
☒	☐	For hierarchical and complex designs, identification of the appropriate level for tests and full reporting of outcomes
☐	☒	Estimates of effect sizes (e.g. Cohen's *d*, Pearson's *r*), indicating how they were calculated

Our web collection on statistics for biologists contains articles on many of the points above.

Software and code

Policy information about availability of computer code

Data collection	No new data were collected for this study; analyses were conducted using publicly available datasets and private datasets provided by collaborators listed in Supplementary Table 1.
Data analysis	Code to calculate all clocks (except for OMICmAge and DNAmEMRAge) are available at https://github.com/HigginsChenLab/methylCIPHER. Code to calculate OMICmAge, DNAmEMRAge, and the associated underlying algorithm can be requested at https://www.trudiagnostic.com/softwarerequest/, at no cost for research purposes. Access is contingent upon the requestor agreeing to a standard, noncommercial software license agreement that confirms the intended use is strictly for academic, nonprofit research and that the requestor will not attempt to reverse engineer or commercially exploit the software. All R codes used to develop and validate EMRAge, DNAmEMRAge and OMICmAge are available at https://github.com/LaskySuLab/OMICmAge/.

For manuscripts utilizing custom algorithms or software that are central to the research but not yet described in published literature, software must be made available to editors and reviewers. We strongly encourage code deposition in a community repository (e.g. GitHub). See the Nature Portfolio guidelines for submitting code & software for further information.

Data

Policy information about availability of data

All manuscripts must include a data availability statement. This statement should provide the following information, where applicable:

- Accession codes, unique identifiers, or web links for publicly available datasets
- A description of any restrictions on data availability
- For clinical datasets or third party data, please ensure that the statement adheres to our policy

No new data were collected for this study. All analyses were performed using publicly available datasets and private datasets obtained from collaborators, as detailed in Supplementary Table 1. Accession numbers or direct download links are provided for all publicly available datasets where possible. Use of private or third-party datasets was granted under data-sharing agreements with the respective collaborators, and access to these datasets may require additional approvals or agreements with the original data providers. No personally identifiable information or new human participant data were collected or generated as part of this study.

Research involving human participants, their data, or biological material

Policy information about studies with human participants or human data. See also policy information about sex, gender (identity/presentation), and sexual orientation and race, ethnicity and racism.

Reporting on sex and gender	Where applicable, the sex of participants was reported based on the source dataset documentation. Analyses involving human cohorts used data as annotated by the original study teams, including sex assigned at birth or DNA methylation calculated sex where available. No additional sex or gender-based analyses were performed in this study unless explicitly stated.
Reporting on race, ethnicity, or other socially relevant groupings	Information on race, ethnicity, or other socially relevant groupings was limited across the datasets used in this study. Where available, such information was included as originally annotated by the source studies and was not reclassified or inferred. Due to inconsistent availability and incomplete representation across cohorts, analyses were not systematically stratified by race or ethnicity. We acknowledge the importance of these variables in health research and highlight the need for more inclusive and standardized demographic reporting in aging and biomarker studies.
Population characteristics	This study utilized data from multiple publicly available and collaborator-shared human cohorts, as outlined in Supplementary Table 1. The included datasets varied in sample size, age distribution, sex composition, and health status. Most participants were middle-aged to older adults, with a majority of datasets enriched for individuals undergoing clinical or longitudinal aging assessments. Due to heterogeneity in source datasets, demographic variables such as sex, race, and ethnicity were included as annotated by original study teams. Where available, these characteristics were used descriptively and/or as covariates in analysis, but were not uniformly present across all cohorts.
Recruitment	No participants were recruited directly for this study. All analyses were conducted using previously collected data from publicly available or collaborator-shared datasets. Recruitment procedures, inclusion/exclusion criteria, and informed consent protocols were determined by the original studies.
Ethics oversight	This study involved only secondary analysis of de-identified data obtained from publicly available sources and collaborator-provided datasets. All original studies received ethics approval from their respective institutional review boards or ethics committees, and informed consent was obtained from participants as described in the source publications. No new human subjects were enrolled, and no additional ethical approval was required for the analyses conducted in this study.

Note that full information on the approval of the study protocol must also be provided in the manuscript.

Field-specific reporting

Please select the one below that is the best fit for your research. If you are not sure, read the appropriate sections before making your selection.

☒ Life sciences ☐ Behavioural & social sciences ☐ Ecological, evolutionary & environmental sciences

For a reference copy of the document with all sections, see nature.com/documents/nr-reporting-summary-flat.pdf

Life sciences study design

All studies must disclose on these points even when the disclosure is negative.

Sample size	This study included secondary analysis of multiple independent datasets, with sample sizes determined by the availability of qualified participants in each source cohort. No statistical methods were used to pre-determine sample sizes, as no new data were collected. Detailed sample sizes for each dataset used in the analysis are provided in Supplementary Table 1. In all cases, sample sizes were sufficient to perform the planned analyses without pooling across incompatible study designs.
Data exclusions	Datasets were included based on the following pre-specified criteria: (1) the study involved an intervention previously hypothesized in the literature to impact longevity or healthspan; (2) DNA methylation (DNAm) data were available at both pre- and post-intervention time points; (3) the tissue of origin for DNAm data was blood, preferably peripheral blood mononuclear cells (PBMCs); and (4) the study met minimum quality thresholds, including an adequate number of individuals in the treatment arm and consistent DNAm profiling methodology across time points. Datasets that did not meet all of these criteria were excluded from analysis.

Replication	This study involved secondary analysis of existing datasets rather than primary experimental replication. Where applicable, findings were validated across multiple independent cohorts that met consistent inclusion criteria (see Supplementary Table 1). All analytic procedures and code used to generate results are available upon reasonable request or will be made publicly available upon publication to facilitate replication and transparency.
Randomization	No randomization was performed as part of this study. All analyses were conducted using previously collected data from observational or interventional studies. Where applicable, randomization procedures were implemented in the original study designs and are described in the corresponding source publications referenced in Supplementary Table 1.
Blinding	No blinding was performed in this study. All analyses were conducted retrospectively using de-identified datasets. In cases where blinding was part of the original study design (e.g., randomized controlled trials), such details are provided in the corresponding source publications referenced in Supplementary Table 1.

Reporting for specific materials, systems and methods

We require information from authors about some types of materials, experimental systems and methods used in many studies. Here, indicate whether each material, system or method listed is relevant to your study. If you are not sure if a list item applies to your research, read the appropriate section before selecting a response.

Materials & experimental systems

n/a	Involved in the study
☒	☐ Antibodies
☒	☐ Eukaryotic cell lines
☒	☐ Palaeontology and archaeology
☒	☐ Animals and other organisms
☒	☐ Clinical data
☒	☐ Dual use research of concern
☒	☐ Plants

Methods

n/a	Involved in the study
☒	☐ ChIP-seq
☒	☐ Flow cytometry
☒	☐ MRI-based neuroimaging

Plants

Seed stocks	*Report on the source of all seed stocks or other plant material used. If applicable, state the seed stock centre and catalogue number. If plant specimens were collected from the field, describe the collection location, date and sampling procedures.*
Novel plant genotypes	*Describe the methods by which all novel plant genotypes were produced. This includes those generated by transgenic approaches, gene editing, chemical/radiation-based mutagenesis and hybridization. For transgenic lines, describe the transformation method, the number of independent lines analyzed and the generation upon which experiments were performed. For gene-edited lines, describe the editor used, the endogenous sequence targeted for editing, the targeting guide RNA sequence (if applicable) and how the editor was applied.*
Authentication	*Describe any authentication procedures for each seed stock used or novel genotype generated. Describe any experiments used to assess the effect of a mutation and, where applicable, how potential secondary effects (e.g. second site T-DNA insertions, mosiacism, off-target gene editing) were examined.*
