# Supplementary material for: Responsiveness of epigenetic aging biomarkers to longevity interventions in humans
Source: Nat Med. 2026 Aug 21;32(9):3477–90. doi: 10.1038/s41591-026-04562-9 (PMC13577923; doi:10.1038/s41591-026-04562-9)
Supplement: Supplementary file 2 [file 41591_2026_4562_MOESM2_ESM.pdf]

nature portfolio

Peer Review File

Epigenetic aging biomarkers are responsive: Insights from 51 longevity interventional studies in humans

Corresponding Author: Dr Raghav Sehgal

This file contains all reviewer reports in order by version, followed by all author rebuttals in order by version.

Version 0:

Reviewer comments:

Reviewer #1

(Remarks to the Author)
I think this article is highly significant for several reasons. It addresses a crucial research question: Are epigenetic biomarkers and clocks responsive to various interventions in humans? While numerous articles describe epidemiological results in human cohorts, this study is distinguished by its analysis of 51 interventional studies. The authors provide a rigorous evaluation of different epigenetic clocks/biomarkers across a sufficient number of trials (n=51), yielding important insights for ranking various biomarkers, interventions, and comparing drugs to supplements. I think this paper represents a significant milestone in the ongoing quest to identify surrogate endpoints for human longevity interventions. Importantly, it addresses whether epigenetic biomarkers can serve as surrogate endpoints in human longevity interventions. The article is both rigorous and well-written.

Comments:

Line 309: Comparison of different clocks. Add a statement explicitly mentioning that PC.GrimAge led to the statistically most significant decrease (p=0.0003) among these second-generation clocks. In my opinion, statistical significance is more important than effect size, which can be compromised by high variability. However, I acknowledge that opinions may vary on this point. Regardless, it is best to include my comment.

Minor. Line 313: I assume these are imputed cell fractions based on methylation? If so, explicitly mention that these were imputed.

Minor. Line 407, 410: Spelling. Why is 'Healthy' capitalized?

Minor. Line 446: Regarding the claim that lavender oil supplements affect organs (LOS decreased Brain 0.29, Immune 0.15, Inflammation 0.19, Kidney 0.2, and Metabolic 0.24). Is this significant after correcting for multiple comparisons? Are these results credible? I am concerned that scientists may dismiss the claim that lavender oil supplements have a significant effect on anything. Unless it is truly significant, I would omit this material to err on the side of caution. The fact that the authors do not claim that LOS affects second-generation clocks enhances my confidence in this particular study and the biomarkers.

Minor: If space is an issue, I would shorten the section 'Study population health status is critical to DNAm biomarker response.' I do not believe this material warrants extensive space since the criterion of working in healthy populations does not seem very important to me. Consider a biomarker that is not significant in either healthy or diseased populations. In this case, the treatment effect will not differ (since it is zero in both) between the two populations.

Major: I would use the freed-up space to add more sentences to lines 457-460 regarding the results of different diets on these epigenetic biomarkers. Readers will undoubtedly be very interested in the results of diet. Which diet is best according to epigenetic clocks? Which diet is best for middle-aged people? Which diet is best for older individuals? Which diet is best for men/women?

Minor: The section on 'Responsiveness of stochastic and causal components' will be difficult for most readers to follow. It may not be of interest to the readership of Nat Medicine. I would shorten or omit this material and use the freed-up space to delve into more interesting topics such as the effects of various diets, possibly exploring differential effects in males/females

or different age groups. Do treatment effects in males agree with those in females? What about treatment effects in middle-aged versus older people?

Minor: Panel 6F. StocP vs PhenoAge. As noted by the authors, PhenoAge is highly subject to technical noise. I suggest using PC.PhenoAge, GrimAge, or other more reliable second-generation clocks.

Major: The section on 'Gen Explainable DNAm biomarkers offer mechanistic insights into responsiveness' will be quite interesting to clinicians and many others. For example, I am struck by the sentence on Line 424: 'Examples of biomarkers with particularly high responsiveness included PCGrimAge components....' It would be beneficial if the authors could comment on whether any of these biomarkers are superior to next-generation clocks such as DunedinPace or PC.GrimAge in interventional studies. Or which of these biomarkers should be measured in longevity trials?

Minor: As pointed out by the authors, the main contribution of the paper is the evaluation of the responsiveness of different epigenetic biomarkers to various interventions. However, since this paper is aimed at a broad audience, the authors should add a sentence(s) to the introduction or discussion pointing to literature where others have evaluated the predictive accuracy of the clocks for mortality/frailty. Which clocks are best for mortality risk prediction? e.g. this text could be added to the Discussion near Line 495 or in the introduction. My reading of the literature suggests that GrimAge outperforms other clocks. A brief Google search found the following, but I am sure I have missed relevant publications. Alternatively, add an unbiased evaluation to the current article in a large data set (NHANES?).

Tiina Föhr, Mortality Associations With DNA Methylation-Based Biological Aging and Physical Functioning Measures Across a 20-Year Follow-up Period, The Journals of Gerontology: Series A, Volume 78, Issue 8, August 2023, Pages 1489–1496, https://doi.org/10.1093/gerona/glad026

Cathal McCrory, The Journals of Gerontology: Series A, Volume 76, Issue 5, May 2021, Pages 741–749, https://doi.org/10.1093/gerona/glaa286

Major: Line 586. Apart from DunedinPACE, also mention PCGrimAge since it led to the statistically most significant results. As mentioned above: statistical significance is more important than effect size, which can be compromised by high variability.

Minor: The Discussion is a bit too long. Some of the material is repetitive. For example, the last paragraph could be shortened.

Major: I am not sure whether the journal will accept the data availability statement 'TranslAGE availability.' I think the data and related software should be deposited in a public database before publication. However, this depends on the journal's policy. Thus, individual-level data, including idat files, annotation data (age, sex, treatment status, etc.), should be distributed. Software scripts that accompany the figures should be released so that others can reproduce the figures.

Reviewer #2

(Remarks to the Author)
In their manuscript Seghal and colleagues explore the association between interventions putatively related to aging and a comprehensive set of DNA methylation clocks (whole blood). The work entails bringing together public data of tens of previously published studies and recalculating DNA methylation clocks. The goal of this analysis as stated by the authors is a relevant one, namely to investigate whether DNAm clocks can act as a surrogate endpoint in clinical trials. The breath of the intervention studies analyzed is of interest and the comprehensive analysis of DNA clocks is important. The work remains exploratory and lacks the rigor to come to conclusions that would significantly contribute to the field. Some limitations include the following issues:
- The work suffers from circular reasoning. The primary question seems to be whether DNAm clocks can capture interventions. But equally often the authors write that the presence or absence of an association with an intervention is informative on the efficacy of the intervention (e.g. senolytics and anti-TNF).
- The authors want to know whether DNA clocks can be used as surrogate markers. A much more clearly defined analysis is necessary to test this. In particular, clinical trials will be focusing on the minimal clinically important difference (MCID). What is this MCID? What is a difference that is meaningful for a DNAm clock to be a useful surrogate endpoint? The authors should be able to work with this key question.
- Now, the authors use a nominal p-value of 0.05 as a measure for an association that would relevant. This is a very minimal criterion, not linking to effect sizes, and bound to lead to overoptimistic interpretations. The authors defend the use of 0.05, as if a single test only was performed (note that even for a single test this threshold is considered to introduce too many false positive findings). The authors evaluate 16 'prominent' DNAm clocks and in total 110 DNAm-based markers. On the one hand the authors argue that the markers measure different aspects but at the same time say they measure similar things (and hence multiple testing correction is not necessary). Both cannot be true. The authors can provide the correlations between all markers and estimate the number of independent tests performed using one of the software packages developed for this issue.
- Note that for the analysis of the 16 'prominent clocks' none of the associations would be significant after correction for (only) 16 tests. Associations are weak and if we follow the results of the authors' DNAm clocks may be poor surrogate endpoints.
- The authors curated public data in a resource. However, this curated resource is not publicly available. The authors 'intend'

to make it available. This does not seem right since they are profiting from other who did make their data publicly available. Likewise, code will not be made publicly available. Given the commercial interests involved transparency is of utmost importance.
- The resource is curated and harmonized but not with respect to DNAm data. It is a missed opportunity that DNAm data were not harmonized using consistent QC and normalization procedures. Note, that throughout the manuscript the use of the terms 'epigenetic clocks' and 'DNAm biomarkers' was not always clear (a smoking predictor seems to be defined as a DNAm biomarker, GrimAge too or is that an epigenetic clock?).
- Batch effects are hugely important in the analysis of DNAm data. However, information on batched was not included in the analysis. In fact, T-tests were performed without including any covariates.
- There is no reference to control (placebo) groups in the manuscript. In particular for the weak associations observed, it is key to establish that such associations were absent in placebo groups.
- The manuscript lacks a clear description of the intervention studies and a rationale why they can be regarded as interventions that would promote longevity. Also, not every study will be of the same quality in terms of design and sample size. Neither will all interventions be equally relevant.
- The manuscript will benefit form a much more complete presentation of associations between DNAm clocks and the various interventions in the main text. Now, many different, ad hoc and difficult to interpret analyses are presented (combining all intervention for example and sub-study analyses).
- In an interesting follow-up analysis, the authors report that associations are present in diseased subjects but not healthy ones. How does this impact on the interpretation of the clocks (including as potential surrogate endpoint)
- Remarkably, Omicmage and DNAmEMRAge are put forward as 'prominent DNAm markers'. The catchy term GenX is reserved for them. Since they are relatively new and the code to produce them is not even available, this is simply impossible. These markers show particularly underwhelming associations with interventions and yet the authors in their discussion claim that they are promising. It is difficult not to link this to the conflicts of interest the authors stated.
- The manuscript can be condensed significantly by increasing the information density and focusing on main analyses.
- Are the authors aware of an intervention where participants were guided to quit smoking? It will be of great interest to see the effects on clocks that are designed to also predict smoking status (e.g Grimage).
- Y other biomarkers. What is Y?
- The first figure discussed is figure 2 (not 1).
- There is a mismatch with the content of figure 1a and the results section describing the figure. Figures 1b and 1c are note even discussed. The purpose of the figures and the interpretation is debatable.
- The legend of figure 3 is of low quality.
- Figure 4e shows more consistency among the dietary interventions then the (more noisy) pharmacological ones, yet the authors conclude the opposite.
- Suppl fig 1: figure content and legend do not match.

Reviewer #3

(Remarks to the Author)
It is still under debate whether interventions impact the epigenetic aging process. The study of Sehgal et al. therefore addresses a very important and timely topic. It provides a comprehensive collection of 51 interventional studies and tested 16 prominent epigenetic clocks into the TranslAGE-Reponse database. Notably, several second generation clocks have coherent response, particularly to pharmacological interventions. While such a comprehensive comparison is intriguing, there are conceptional difficulties in numerical comparison of the number of studies - with very different designs, overlapping epigenetic biomarkers, and possible confounding changes in clinical parameters. Thus, I feel that the findings of this comparison (which of course could not be validated on independent sets of studies) should be more critically discussed. I have the following specific concerns:

1) Unfortunately, TranslAGE is apparently not easily accessible to other researchers. Furthermore, about half of the studies were generated by TrueDiagnostics and are apparently not open access. The authors should better clarify how they "intend for TranslAGE to become available as a resource to the geroscience community."

2) If 110 DNAm biomarkers are tested, additional adjustment for multiple testing may be required (particularly if the threshold is only set to 0.05).

3) Different types of interventional studies have been pooled – with different age ranges, treatment regimen, duration, etc. I understand that pooling of effect sizes may give clearer results, but from a biological perspective this may be a bit irritating. I would suggest to rather not show this.

4) The different DNAm biomarkers are not independent. There is probably a significant overlap in the CpGs between these signatures that needs to be specified and discussed.

5) Gen 2+ clocks have been trained based on additional age-associated parameters. While I fully agree that this may be valuable to quantify aspects of biological aging, there is also the danger that effects are largely attributed to changes in these parameters (e.g. to cellular composition or CRP). For example. GrimAgeV2 was also trained on c-reactive protein – a relevant parameter associated with effect size (Fig 6). Yet, interventions that change CRP do not necessarily impact the aging process but rather signify health status and inflammation (PMID: 38692281). Thus, caution needs to be taken when using Gen 2+ clocks to investigate effects of interventions on the aging process. I believe this is a general conceptual problem that needs to be discussed.

Minor:
6) It would be good to mention the total number of samples analyzed. Furthermore, additional information on the study design of the 51 studies should be provided in the supplemental table (particularly for those studies that are apparently not yet published). Please include also links to the publications, if available.

7) P3: “The first epigenetic clocks” are not cited here (perhaps rephrase, “relatively early epigenetic clocks”.)

8) P4: Please include a reference that these clocks are “… collectively known as Generation 2+ clocks”. I believe they are more commonly referred to as second generation clocks.

9) Last section of the introduction “… and Y other DNAm biomarkers”.

10) Are there differences by sex? And of the other clinical covariates (as also suggested in the comment that still remained in the word file)

11) In figure 3A (label in the legend is wrong) is the standard deviation indicated? Could it be that the effect size is largely affected by few individual studies?

12) The effect size is presumably very small. If effects on epigenetic age predictions are only marginal (i.e. few days), then this should be discussed.

13) I am not sure if it has been shown that DNAm biomarkers preferentially capture stochastic epigenetic noise – while this is conceivable and supported by some models, it is still not proven. Furthermore, their finding on this aspect was only shown for StocH vs StocP and no other models.

14) The discussion repeats some of the results. Instead, it would be valuable to critically discuss the limitations of this study.

15) Are there systematic effects of interventions on cell count predictions?

16) Please provide reference for MethylCIPHER 2.0

17) Perhaps figure 4c might be represented as a heatmap?

18) There are several typos and inconsistencies that should be corrected.

Version 1:

Reviewer comments:

Reviewer #1

(Remarks to the Author)
I thank the authors for thoroughly addressing my comments. I have no further comments. Congratulations on this important study.

Reviewer #2

(Remarks to the Author)
While it was obvious from my first review that I am less enthusiastic about the work than the other reviewers and editor, I appreciate the authors' responsiveness, effort to make the results/data publicly available (as I understand this process is ongoing), and the careful additions to the discussion. Two questions remain for me:
1. The authors discuss correction for multiple testing in their rebuttal and manuscript, but do not actually implement it. The authors argue (in line with my original remark) that taking the correlation between tests into account (instead of performing Bonferroni correction) is a way forward. I also agree that taking the number of the principal components capturing 90% of the total variance may be defendable. However, the authors continue to state that the use (and also report) on findings P<0.05. I believe this should be changed and only findings significant after accounting for multiple testing should be reported to prevent over-interpretation with respect to the utility of epigenetic clocks. (On the side: I still do not understand why T tests instead of multivariable analyses were done to account for the obvious influence of covariates)
2. The authors state that 'The other two clocks, OMICmAge and DNAmEMRage, are proprietary algorithms. These are available commercially through TruDiagnostic, and we have clarified this in the text and ensured that citations are included.' I strongly believe this should have the consequence that both clocks cannot be included in the paper. It goes against open science and reproducibility. The clock-field already suffers from potential conflicts of interests (which for clinical studies are well-known to influence outcomes of scientific studies). The fact that the clocks cannot be used by the field turn a scientific paper into an advertisement of a commercial product.

Reviewer #3

(Remarks to the Author)
As indicated previously, this integrative analysis of 51 interventional studies provides an important and timely overview of aging biomarkers. In particular, the observed deviations in predictions are highly informative for the field, as they help delineate the current strength and limitations of epigenetic clocks. I appreciate that the revised manuscript now includes a more explicit discussion of these limitations, especially the point that generation 2+ clocks may primarily capture health-related parameters rather than the aging trajectory per se. While such measures are undoubtedly relevant for health outcomes, they should not be equated with biological rejuvenation.

Overall, the authors have carefully revised the manuscript and addressed the majority of my concerns. Although not all suggestions were fully implemented, their explanations are reasonable and scientifically ok. The authors also state that all publicly available datasets used in TranslAGE will be fully accessible upon publication and that they are working toward a compliant data-sharing pathway for the TruDiagnostic datasets. I assume that this will be finalized prior to publication. In this regard, Supplementary Table 1 is already a helpful step toward greater transparency.

Open Access This Peer Review File is licensed under a Creative Commons Attribution 4.0 International License, which permits use, sharing, adaptation, distribution and reproduction in any medium or format, as long as you give appropriate credit to the original author(s) and the source, provide a link to the Creative Commons license, and indicate if changes were made.
In cases where reviewers are anonymous, credit should be given to 'Anonymous Referee' and the source.
The images or other third party material in this Peer Review File are included in the article’s Creative Commons license, unless indicated otherwise in a credit line to the material. If material is not included in the article’s Creative Commons license and your intended use is not permitted by statutory regulation or exceeds the permitted use, you will need to obtain permission directly from the copyright holder.
To view a copy of this license, visit https://creativecommons.org/licenses/by/4.0/

Reviewers' Comments:

Reviewer #1 (Remarks to the Author):

I think this article is highly significant for several reasons. It addresses a crucial research question: Are epigenetic biomarkers and clocks responsive to various interventions in humans? While numerous articles describe epidemiological results in human cohorts, this study is distinguished by its analysis of 51 interventional studies. The authors provide a rigorous evaluation of different epigenetic clocks/biomarkers across a sufficient number of trials (n=51), yielding important insights for ranking various biomarkers, interventions, and comparing drugs to supplements. I think this paper represents a significant milestone in the ongoing quest to identify surrogate endpoints for human longevity interventions. Importantly, it addresses whether epigenetic biomarkers can serve as surrogate endpoints in human longevity interventions. The article is both rigorous and well-written.

Thank you for your thoughtful and encouraging review. We appreciate your recognition of the study's significance in evaluating the responsiveness of epigenetic biomarkers across 51 human interventions. Your comments affirm our aim to rigorously assess their potential as surrogate endpoints in longevity trials. We're grateful for your support and are pleased the manuscript was clear and impactful.

Comments:

Line 309: Comparison of different clocks. Add a statement explicitly mentioning that PC.GrimAge led to the statistically most significant decrease (p=0.0003) among these second-generation clocks. In my opinion, statistical significance is more important than effect size, which can be compromised by high variability. However, I acknowledge that opinions may vary on this point. Regardless, it is best to include my comment.

Thank you for this valuable suggestion. We have revised the manuscript to explicitly note that PC-GrimAge exhibited the most statistically significant decrease among the second-generation clocks (p = 0.0003). We agree that statistical significance is a critical consideration, especially given potential variability in effect size, and we have incorporated your perspective accordingly.

Minor. Line 313: I assume these are imputed cell fractions based on methylation? If so, explicitly mention that these were imputed.

Thank you for pointing this out. We have clarified the text to explicitly state that these are imputed cell fractions derived from DNA methylation data.

Minor. Line 407, 410: Spelling. Why is 'Healthy' capitalized?

Thank you for catching this. We have corrected the capitalization, "healthy" is now lowercase, as it is not a proper noun in this context.

Minor. Line 446: Regarding the claim that lavender oil supplements affect organs (LOS decreased Brain 0.29, Immune 0.15, Inflammation 0.19, Kidney 0.2, and Metabolic 0.24). Is this significant after correcting for multiple comparisons? Are these results credible? I am concerned that scientists may dismiss the claim that lavender oil supplements have a significant effect on anything. Unless it is truly significant, I would omit this material to err on the side of caution. The fact that the authors do not claim that LOS affects second-generation clocks enhances my confidence in this particular study and the biomarkers.

Thank you for this thoughtful comment. We agree that extraordinary claims require careful scrutiny. Therefore, we have removed this material from the main text and refrained from highlighting it in our conclusions.

We appreciate your note regarding the cautious treatment of this finding and your confidence in the study's overall approach.

Minor: If space is an issue, I would shorten the section 'Study population health status is critical to DNAm biomarker response.' I do not believe this material warrants extensive space since the criterion of working in healthy populations does not seem very important to me. Consider a biomarker that is not significant in either healthy or diseased populations. In this case, the treatment effect will not differ (since it is zero in both) between the two populations.

Thank you for this suggestion. We appreciate your point that differences in biomarker responsiveness across healthy and diseased populations may not always be informative, particularly when no treatment effect is observed in either group. In response, we have shortened the section titled "Study population health status is critical to DNAm biomarker response" to streamline the discussion.

At the same time, we believe it is important to clarify why health status can matter. Clinical trials that target healthy versus diseased populations often have fundamentally different aims: it is intuitively easier to slow down already-accelerated aging than to slow down normative aging. This is why some researchers preferentially study diseased populations, where the likelihood of observing a measurable effect is higher. Moreover, the populations in which biomarkers were originally trained also shape their sensitivity. For example, DunedinPACE was developed in a younger, healthier cohort, and may therefore be particularly responsive in healthy populations. Our results align with this expectation, underscoring why study population health status should not be ignored, even if the emphasis in our discussion is now more balanced.

Major: I would use the freed-up space to add more sentences to lines 457-460 regarding the results of different diets on these epigenetic biomarkers. Readers will undoubtedly be very interested in the results of diet. Which diet is best according to epigenetic clocks? Which diet is best for middle-aged people? Which diet is best for older individuals? Which diet is best for

men/women?

We appreciate the reviewer's excellent suggestion and agree that the effects of diet on epigenetic biomarkers are of substantial interest to readers. In response, we have cleaned and clarified the corresponding heatmap and included it as Supplementary Figure 2 to illustrate the comparative effects of different dietary interventions across multiple epigenetic clocks.

We fully recognize the importance of the reviewer's questions regarding which diets and subgroups (e.g., by age or sex) show the most favorable associations. To address these questions more comprehensively, we are preparing a separate, dedicated manuscript focusing specifically on dietary and other lifestyle interventions including distinct exercise regimens accompanied by a rigorous stratified meta-analysis. We felt that such in-depth analyses were beyond the scope of the current paper, which aims to establish generalizable intervention responsiveness across biomarker classes.

Minor: The section on 'Responsiveness of stochastic and causal components' will be difficult for most readers to follow. It may not be of interest to the readership of Nat Medicine. I would shorten or omit this material and use the freed-up space to delve into more interesting topics such as the effects of various diets, possibly exploring differential effects in males/females or different age groups. Do treatment effects in males agree with those in females? What about treatment effects in middle-aged versus older people?

We thank the reviewer for this thoughtful comment and fully agree that topics such as diet, sex differences, and age-specific effects are of great interest. As noted, we are preparing a separate manuscript that will explore those dimensions in detail.

We have, however, chosen to retain the section on the "Responsiveness of stochastic and causal components" because it addresses a foundational question essential to biomarker interpretation namely, whether intervention-induced changes reflect true biological shifts or are driven by stochastic variability. We have revised and shortened the section for clarity and added text clarifying that our analysis quantifies the proportion of variance in Horvath1 and PhenoAge attributable to stochastic versus non-stochastic components. This allows us to test whether interventions preferentially influence systematic (causal) versus random (stochastic) methylation changes. While prior studies (e.g., Meyer and Schumacher) have argued that stochastic changes can be biologically meaningful, our findings suggest that intervention-associated shifts predominantly affect the non-stochastic component, aligning more closely with the framework proposed by Tarkhov et al. These revisions strengthen the conceptual clarity and translational relevance of this section.

Minor: Panel 6F. StocP vs PhenoAge. As noted by the authors, PhenoAge is highly subject to technical noise. I suggest using PC.PhenoAge, GrimAge, or other more reliable second-generation clocks.

Thank you for this thoughtful suggestion. We agree that PC-based clocks such as PC-PhenoAge

tend to have improved technical reliability over their original counterparts. However, in this case, we intentionally used PhenoAge rather than PC-PhenoAge to allow for a direct, 1-to-1 comparison with its corresponding stochastic component (StocP), which was trained using the original PhenoAge algorithm.

It's important to note that PCPhenoAge is not a simple reformulation but a distinct algorithm with new loadings, trained on a larger dataset. As a result, the outputs of PhenoAge and PC-PhenoAge, while nominally related, are not directly comparable for this specific analysis. Using PC-PhenoAge would introduce an inconsistency in the modeling framework underlying StocP.

Major: The section on 'Gen Explainable DNAm biomarkers offer mechanistic insights into responsiveness' will be quite interesting to clinicians and many others. For example, I am struck by the sentence on Line 424: 'Examples of biomarkers with particularly high responsiveness included PCGrimAge components....' It would be beneficial if the authors could comment on whether any of these biomarkers are superior to next-generation clocks such as DunedinPace or PC.GrimAge in interventional studies. Or which of these biomarkers should be measured in longevity trials?

In our analysis, several principal component (PC) biomarkers, including PC CystatinC, PC ADM, and PC PACKYRS, showed stronger and more reproducible responsiveness to interventions than many composite clocks. Because these biomarkers are pathway proximal, they often capture earlier and larger shifts in inflammation, cardiorenal function, and metabolic signaling. For longevity trials, incorporating a focused panel of PC biomarkers, particularly those anchored in inflammatory and cardiorenal axes, may provide higher sensitivity to change and greater mechanistic interpretability than integrative measures alone.

An additional consideration is that integrative biomarkers may "average out" effects across components. For example, if one PC biomarker increases while another decreases, the composite measure may show little net change. Similarly, if only a single PC biomarker shifts, this effect may be diluted when combined with others. This averaging could explain why certain composite clocks appear less responsive in our analysis.

These findings are also consistent with the idea that interventions targeting specific pathways would be expected to influence specific biomarkers. Highlighting these pathway-focused effects aligns with emerging frameworks such as SystemsAge, which emphasizes organ- and system-level specificity.

We write in the Discussion:

"We further demonstrated that Gen X "explainable" DNAm biomarkers, those targeting specific physiological systems, can provide mechanistic insights into which organ systems are affected. For example, smoking cessation selectively reduced lung system scores, while metformin influenced inflammatory, brain, and metabolic pathways. These system-level scores offer a complementary approach to global clocks and may guide the development of targeted aging

therapies. In our analysis, we also observed several principal component (PC) biomarkers, including PC CystatinC, PC ADM, PC PACKYRS, showed stronger and more reproducible responsiveness to interventions than many composite clocks. Because these biomarkers are pathway proximal, they often capture earlier and larger shifts in inflammation, cardio renal function, and metabolic signaling.48–51 For longevity trials, incorporating a focused panel of PC biomarkers particularly those anchored in inflammatory and cardio renal axes such as PC CystatinC, PC ADM, and PC PACKYRS may provide higher sensitivity to change and greater mechanistic interpretability than integrative measures alone.52"

Minor: As pointed out by the authors, the main contribution of the paper is the evaluation of the responsiveness of different epigenetic biomarkers to various interventions. However, since this paper is aimed at a broad audience, the authors should add a sentence(s) to the introduction or discussion pointing to literature where others have evaluated the predictive accuracy of the clocks for mortality/frailty. Which clocks are best for mortality risk prediction? e.g. this text could be added to the Discussion near Line 495 or in the introduction. My reading of the literature suggests that GrimAge outperforms other clocks. A brief Google search found the following, but I am sure I have missed relevant publications. Alternatively, add an unbiased evaluation to the current article in a large data set (NHANES?).

Tiina Föhr, Mortality Associations With DNA Methylation-Based Biological Aging and Physical Functioning Measures Across a 20-Year Follow-up Period, The Journals of Gerontology: Series A, Volume 78, Issue 8, August 2023, Pages 1489–1496, https://doi.org/10.1093/gerona/glad026

Cathal McCrory, The Journals of Gerontology: Series A, Volume 76, Issue 5, May 2021, Pages 741–749,

Thank you for highlighting this important point. In response, we have added the above citations and Biolearn citation by Ying et al to the discussion with the following text

"Epigenetic clocks, falling under the umbrella of DNA methylation (DNAm) biomarkers, have long been proposed as surrogate endpoints for longevity clinical trials.2,34 While their prognostic value (i.e., association with morbidity and mortality) is well established, with clocks such as GrimAge and GrimAge2 showing the highest mortality association and healthspan prediction(Föhr et al. 2023; Ying et al. 2025; McCrory et al. 2021), their responsiveness to interventions has not been systematically tested across multiple trials and biomarkers."

Major: Line 586. Apart from DunedinPACE, also mention PCGrimAge since it led to the statistically most significant results. As mentioned above: statistical significance is more important than effect size, which can be compromised by high variability.

Thank you for highlighting this important point. In response, we have updated the text to include PC-GrimAge alongside DunedinPACE, noting that PC-GrimAge showed the most statistically

significant responsiveness (p = 0.0003) among the clocks evaluated. We also acknowledge your point that statistical significance provides a more robust indicator of responsiveness in the presence of variable effect sizes.

Minor: The Discussion is a bit too long. Some of the material is repetitive. For example, the last paragraph could be shortened.

Thank you for this helpful suggestion. We have carefully revised the Discussion to reduce length and remove redundancies. In particular, we have shortened the final paragraph to avoid repetition while preserving the key conclusions and future directions.

Major: I am not sure whether the journal will accept the data availability statement 'TranslAGE availability.' I think the data and related software should be deposited in a public database before publication. However, this depends on the journal's policy. Thus, individual-level data, including idat files, annotation data (age, sex, treatment status, etc.), should be distributed. Software scripts that accompany the figures should be released so that others can reproduce the figures.

Thank you for raising this important point. To address this, we have already started constructing the platform for making the data publicly available using the TranslAGE.io website (https://www.translage.io/responsive). We continue to work on this database on the TranslAGE-Response resource to make it publicly available including all curated beta value matrices and associated metadata (e.g., age, sex, intervention group, timepoint) from the publicly sourced studies. In cases where original datasets required data use agreements, we will provide instructions and references for independent access. This includes TruDiagnostic datasets, where harmonized datasets will be returned to TruDiagnostic so that anyone may apply for them through TruDiagnostic.

Additionally, all code used for biomarker calculation can be performed using MethylCIPHER package (https://github.com/HigginsChenLab/methylCIPHER).

Additionally, all analysis scripts and figure-generation code will be released to support full reproducibility. The Data Availability section will be updated with repository links upon final acceptance.

Reviewer #2 (Remarks to the Author):

In their manuscript Seghal and colleagues explore the association between interventions putatively related to aging and a comprehensive set of DNA methylation clocks (whole blood). The work entails bringing together public data of tens of previously published studies and recalculating DNA methylation clocks. The goal of this analysis as stated by the authors is a relevant one, namely to investigate whether DNAm clocks can act as a surrogate endpoint in

clinical trials. The breath of the intervention studies analyzed is of interest and the comprehensive analysis of DNA clocks is important. The work remains exploratory and lacks the rigor to come to conclusions that would significantly contribute to the field. Some limitations include the following issues:

- The work suffers from circular reasoning. The primary question seems to be whether DNAm clocks can capture interventions. But equally often the authors write that the presence or absence of an association with an intervention is informative on the efficacy of the intervention (e.g. senolytics and anti-TNF).

Thank you for highlighting this important point. We acknowledge the potential concern about circular reasoning. To clarify, the primary aim of our study is to evaluate the responsiveness of DNAm biomarkers, not to establish the efficacy of interventions themselves. When we note that certain interventions (e.g., anti-TNF therapies) elicit strong DNAm responses, we interpret this as evidence of the biomarker's responsiveness, not a definitive statement about clinical efficacy.

However, we agree that in several places the language may have inadvertently implied that DNAm response equates to intervention efficacy. We have revised the manuscript to clarify that these associations are used to evaluate biomarker performance, not to validate or rank the interventions themselves. Our focus remains on assessing which biomarkers robustly and consistently register change across diverse interventional contexts.

- The authors want to know whether DNA clocks can be used as surrogate markers. A much more clearly defined analysis is necessary to test this. In particular, clinical trials will be focusing on the minimal clinically important difference (MCID). What is this MCID? What is a difference that is meaningful for a DNAm clock to be a useful surrogate endpoint? The authors should be able to work with this key question.

Thank you for this insightful comment. We fully agree that establishing DNA methylation clocks as surrogate endpoints ultimately requires anchoring their changes to clinically meaningful outcomes, such as the minimal clinically important difference (MCID). At present, the field lacks an empirically defined MCID for DNAm biomarkers, which presents a key barrier to their adoption in clinical trials.

Our current study focuses on an earlier step in the validation process: evaluating responsiveness across interventions. While this does not directly establish MCID thresholds, it helps identify which biomarkers reliably detect change, a necessary precursor to linking those changes to clinical endpoints.

We have added a statement to the Discussion acknowledging this limitation and emphasizing that defining an MCID for DNAm biomarkers is a critical next step. We agree that future work should aim to correlate changes in DNAm clocks with improvements in functional, physiological, or disease-specific outcomes to establish such thresholds and support regulatory acceptance as surrogate endpoints.

*“**Limitations of this study should be acknowledged.** First, we pooled effect sizes across heterogeneous interventional studies that differed substantially in participant age ranges, intervention modalities, durations, and study designs. While this heterogeneity limits biological specificity and precludes interpretation of pooled effects as mechanistic equivalence, our intent was descriptive rather than causal: to characterize broad patterns of DNAm biomarker responsiveness and prioritize clocks that show consistent sensitivity to change. Accordingly, biological interpretation is emphasized within intervention classes, which are analyzed separately throughout the manuscript. Second, variability in study quality, sample size, and intervention relevance is an inherent limitation; however, this diversity provides a wide distribution of effect sizes that is informative for benchmarking biomarker behavior rather than evaluating individual intervention efficacy. Third, lack of harmonized preprocessing and batch correction across studies may affect comparability, and future updates to TranslAGE-Response will incorporate such adjustments where metadata permit. Fourth, interpretation of Generation 2+ clocks requires caution, as their training on composite risk factors (e.g., C-reactive protein in GrimAgeV2) means observed responsiveness may reflect modulation of intermediate health or inflammatory states rather than direct effects on aging biology per se. **Fifth, the absence of an empirically defined minimal clinically important difference (MCID) for DNAm clocks remains a key barrier to their use as surrogate endpoints; future work must anchor clock changes to clinically meaningful functional, physiological, or disease-specific outcomes.** Finally, although standardized effect sizes may appear modest, for widely used clocks such as Horvath, Hannum, PhenoAge, and GrimAge, where the standard deviation of epigenetic age deviation is typically on the order of ~4–6 years, an effect size of ~0.2 corresponds to an approximate one-year shift in epigenetic age, indicating that the observed changes are not biologically trivial.”*

- Now, the authors use a nominal p-value of 0.05 as a measure for an association that would relevant. This is a very minimal criterion, not linking to effect sizes, and bound to lead to overoptimistic interpretations. The authors defend the use of 0.05, as if a single test only was performed (note that even for a single test this threshold is considered to introduce too many false positive findings). The authors evaluate 16 ‘prominent’ DNAm clocks and in total 110 DNAm-based markers. On the one hand the authors argue that the markers measure different aspects but at the same time say they measure similar things (and hence multiple testing correction is not necessary). Both cannot be true. The authors can provide the correlations between all markers and estimate the number of independent tests performed using one of the software packages developed for this issue.

- Note that for the analysis of the 16 ‘prominent clocks’ none of the associations would be significant after correction for (only) 16 tests. Associations are weak and if we follow the results of the authors’ DNAm clocks may be poor surrogate endpoints.

We thank the reviewer for these thoughtful comments. In the revised manuscript, we have clarified our statistical approach by adopting an independent testing framework following Li et al. (2011)

as described in the methods section. For analysis, rather than assuming that all biomarkers tested are independent, we apply principal component analysis (PCA) and count the number of independent components needed to explain 90% of the variance. This value, M_e, represents the effective number of independent tests for that specific analysis.

Because epigenetic biomarkers and interventions are often highly correlated, M_e is typically much smaller than the raw number of tests. Thus, using a nominal threshold of $p < 0.05$ approximates a Bonferroni-style correction based on M_e rather than on the full set of comparisons. To ensure transparency, we provide the estimated M_e and bonferroni thresholds based on M_e values for each analysis in the Supplement Table 23. These multiple tested corrected p-values have been shown in specific figures and mentioned in the text as well.

Finally, we emphasize that our interpretation does not rest solely on nominal significance. We focus on the consistency of effect sizes across interventions, which, together with the M_e-based adjustment, reduces the risk of false positives while maintaining sensitivity to biologically meaningful signals.

- The authors curated public data in a resource. However, this curated resource is not publicly available. The authors 'intend' to make it available. This does not seem right since they are profiting from other who did make their data publicly available. Likewise, code will not be made publicly available. Given the commercial interests involved transparency is of utmost importance.

Thank you for this important point. We fully agree that transparency is essential, particularly when working with publicly shared data, and that access to curated resources and analysis code is a foundational part of scientific integrity and reproducibility.

To address this, we have already started constructing the platform for making the data publicly available using the [TranslAGE.io](https://www.translage.io/responsive) website (https://www.translage.io/responsive). We continue to work on this database on the TranslAGE-Response resource to make it publicly available including all curated beta value matrices and associated metadata (e.g., age, sex, intervention group, timepoint) from the publicly sourced studies. In cases where original datasets required data use agreements, we will provide instructions and references for independent access.

Additionally, all code used for biomarker calculation can be performed using MethylCIPHER package (https://github.com/HigginsChenLab/methylCIPHER).

We appreciate your emphasis on this issue and are fully committed to open and reproducible research, regardless of any commercial interests involved.

- The resource is curated and harmonized but not with respect to DNAm data. It is a missed opportunity that DNAm data were not harmonized using consistent QC and normalization procedures. Note, that throughout the manuscript the use of the terms 'epigenetic clocks' and 'DNAm biomarkers' was not always clear (a smoking predictor seems to be defined as a DNAm

biomarker, GrimAge too or is that an epigenetic clock?).

Thank you for this valuable feedback. We acknowledge that while our primary curation and harmonization efforts focused on study-level metadata, intervention types, and sample annotations, we did not apply a fully standardized quality control (QC) and normalization pipeline across all DNAm datasets. This was a conscious decision due to the heterogeneity in available raw data formats (e.g., some studies lacked IDAT files) and varying preprocessing pipelines already applied in public repositories. However, we agree that harmonizing DNAm data across studies would improve comparability, and we have noted this as a limitation in the revised manuscript in discussion.

*"**Limitations of this study should be acknowledged.** First, we pooled effect sizes across heterogeneous interventional studies that differed substantially in participant age ranges, intervention modalities, durations, and study designs. While this heterogeneity limits biological specificity and precludes interpretation of pooled effects as mechanistic equivalence, our intent was descriptive rather than causal: to characterize broad patterns of DNAm biomarker responsiveness and prioritize clocks that show consistent sensitivity to change. Accordingly, biological interpretation is emphasized within intervention classes, which are analyzed separately throughout the manuscript. Second, variability in study quality, sample size, and intervention relevance is an inherent limitation; however, this diversity provides a wide distribution of effect sizes that is informative for benchmarking biomarker behavior rather than evaluating individual intervention efficacy. **Third, lack of harmonized preprocessing and batch correction across studies may affect comparability, and future updates to TranslAGE-Response will incorporate such adjustments where metadata permit.** Fourth, interpretation of Generation 2+ clocks requires caution, as their training on composite risk factors (e.g., C-reactive protein in GrimAgeV2) means observed responsiveness may reflect modulation of intermediate health or inflammatory states rather than direct effects on aging biology per se. Fifth, the absence of an empirically defined minimal clinically important difference (MCID) for DNAm clocks remains a key barrier to their use as surrogate endpoints; future work must anchor clock changes to clinically meaningful functional, physiological, or disease-specific outcomes. Finally, although standardized effect sizes may appear modest, for widely used clocks such as Horvath, Hannum, PhenoAge, and GrimAge, where the standard deviation of epigenetic age deviation is typically on the order of ~4–6 years, an effect size of ~0.2 corresponds to an approximate one-year shift in epigenetic age, indicating that the observed changes are not biologically trivial."*

We are preparing a separate manuscript on systematic preprocessing of DNAm data and the effects on clock performance including intervention effects. Importantly, prior work has shown that DNAm age acceleration estimates do not vary substantially across different preprocessing methods, and we would not expect preprocessing to meaningfully affect longitudinal changes in epigenetic age given that any systematic shifts due to preprocessing would affect both baseline and follow-up (McEwen et al., 2018). Nonetheless, it would be valuable to demonstrate this

empirically in at least one dataset, and we plan to explore this in future iterations. For future releases of TranslAGE-Response, we also intend to implement a unified normalization and QC framework using raw data wherever available.

Regarding terminology: you are absolutely right that our use of "epigenetic clocks" and "DNAm biomarkers" could be more precise. We have revised the manuscript to clarify that:

- ***Epigenetic clocks** refer to composite DNAm algorithms trained to predict chronological age, biological age, or time-to-event outcomes (e.g., Horvath1, PhenoAge, GrimAge, DunedinPACE).*

- ***DNAm biomarkers**, in our framework, is a broader term that includes both clocks and individual CpG-based surrogates for physiological traits (e.g., DNAm-predictors of smoking, C-reactive protein, leptin). We use this more commonly to include both Epigentic clocks and other epigenetic based biomarkers.*

We have now defined both terms clearly in the Introduction and ensured consistent usage throughout the manuscript.

- Batch effects are hugely important in the analysis of DNAm data. However, information on batched was not included in the analysis. In fact, T-tests were performed without including any covariates.

Thank you for raising this important point. We fully recognize the relevance of batch effects in DNAm data analysis. While our primary approach used within-study paired t-tests, which control for inter-individual variability and between-study batch effects, we acknowledge that explicit within-study batch correction (e.g., plate or array ID) was not applied, as batch metadata were inconsistently reported across public datasets.

That said, to assess whether technical or demographic confounders may influence DNAm biomarker responsiveness, we systematically tested associations between effect sizes and eight study-level variables:

1. *Study population health status (healthy vs. disease),*
2. *Sample size,*
3. *Intervention duration,*
4. *Mean age,*

5. *Age standard deviation,*
6. *Minimum age,*
7. *Age range, and*
8. *Percent female.*

These analyses (described in Figure 5A and Supplementary Table 13) allowed us to identify population-level covariates that may bias biomarker responsiveness. Among these, population health status emerged as the most consistent factor. While this does not replace batch-level correction at the sample level, it provides a first-pass assessment of structured confounding.

We now explicitly acknowledge in the Discussion that lack of batch correction is a limitation and plan to incorporate such adjustments in future updates to TranslAGE-Response, contingent on metadata availability.

*"**Limitations of this study should be acknowledged.** First, we pooled effect sizes across heterogeneous interventional studies that differed substantially in participant age ranges, intervention modalities, durations, and study designs. While this heterogeneity limits biological specificity and precludes interpretation of pooled effects as mechanistic equivalence, our intent was descriptive rather than causal: to characterize broad patterns of DNAm biomarker responsiveness and prioritize clocks that show consistent sensitivity to change. Accordingly, biological interpretation is emphasized within intervention classes, which are analyzed separately throughout the manuscript. **Second, variability in study quality, sample size, and intervention relevance is an inherent limitation; however, this diversity provides a wide distribution of effect sizes that is informative for benchmarking biomarker behavior rather than evaluating individual intervention efficacy. Third, lack of harmonized preprocessing and batch correction across studies may affect comparability, and future updates to TranslAGE-Response will incorporate such adjustments where metadata permit.** Fourth, interpretation of Generation 2+ clocks requires caution, as their training on composite risk factors (e.g., C-reactive protein in GrimAgeV2) means observed responsiveness may reflect modulation of intermediate health or inflammatory states rather than direct effects on aging biology per se. Fifth, the absence of an empirically defined minimal clinically important difference (MCID) for DNAm clocks remains a key barrier to their use as surrogate endpoints; future work must anchor clock changes to clinically meaningful functional, physiological, or disease-specific outcomes. Finally, although standardized effect sizes may appear modest, for widely used clocks such as Horvath, Hannum, PhenoAge, and GrimAge, where the standard deviation of epigenetic age deviation is typically on the order of ~4–6 years, an effect size of ~0.2 corresponds to an approximate one-year shift in epigenetic age, indicating that the observed changes are not biologically trivial."*

- There is no reference to control (placebo) groups in the manuscript. In particular for the weak associations observed, it is key to establish that such associations were absent in placebo groups.

Thank you for raising this important point. We fully agree that the inclusion and analysis of placebo or control groups is critical for interpreting the specificity of DNAm biomarker responses, particularly when effect sizes are modest.

To address this, we included placebo/control arms in our analysis where available and explicitly present these results in ***Figure 3A****. As shown, the control arms demonstrated minimal to no changes across DNAm clocks, supporting the conclusion that the observed effects in treatment groups are not attributable to technical drift or time alone. We have now added clarifying language in the Results and Figure 3 legend to make this explicit.*

We appreciate your suggestion and agree this strengthens the rigor of the interpretation.

- The manuscript lacks a clear description of the intervention studies and a rationale why they can be regarded as interventions that would promote longevity. Also, not every study will be of the same quality in terms of design and sample size. Neither will all interventions be equally relevant.

Thank you for this important point. We agree that not all intervention studies are of equal quality or relevance, and that sample size and study design vary considerably. However, our aim in this analysis was not to evaluate the efficacy of individual interventions per se, but rather to examine general patterns of biomarker responsiveness across a diverse set of studies. In this context, both high-quality studies of interventions likely to affect longevity and lower-quality studies of less relevant interventions are informative, as together they provide a broader range of effect sizes and p-values. This variability allows us to better characterize the general behavior of DNAm biomarkers under different conditions. That being said for the purposes of transparency we have added all such necessary information in Supplementary Table 1. For example the following columns are added in Supplementary Table 1: Intervention, GEO ID or Dataset ID, , link to main publication (PMID), Sample Type, Array Study ID (Or study info source).Study Design: Primary Purpose. Study Design: Allocation. Study Design: Interventional Model. Study Design: Masking, Inclusion/ Exclusion Criteria, Intervention Regimen, MoA, Intervention Category, Condition, Sex Distribution, Sample count, Age Range, Length of intervention(tmax) and more. We have also made a point of this in discussion in the limitations section.

- The manuscript will benefit form a much more complete presentation of associations between DNAm clocks and the various interventions in the main text. Now, many different, ad hoc and difficult to interpret analyses are presented (combining all intervention for example and sub-study analyses).

All associations between DNAm clocks and interventions, including effect sizes, p-values, intervention types, study-level metadata, and stratified analyses, are comprehensively provided in the Supplementary Materials (specifically, Supplementary Tables 5–15).

The main text was intentionally streamlined to highlight key findings without overwhelming the reader with redundant data. We believe the current structure strikes a balance between clarity and completeness. However, in response to your comment, we have added cross-references in

the main text to direct readers more explicitly to the relevant supplementary tables where the full set of associations can be reviewed.

- In an interesting follow-up analysis, the authors report that associations are present in diseased subjects but not healthy ones. How does this impact on the interpretation of the clocks (including as potential surrogate endpoint)

Thank you for highlighting this point. We agree that the differential responsiveness of DNAm biomarkers in diseased versus healthy populations has important implications for their interpretation and utility as surrogate endpoints. As discussed in the manuscript, we found that several Generation 2+ clocks (e.g., PCPhenoAge, SystemsAge) showed greater responsiveness in disease populations, likely due to higher baseline dysregulation or greater biological plasticity in these individuals.

However, this pattern was not universal, notably, DunedinPACE demonstrated responsiveness in both healthy and diseased populations, suggesting their broader applicability. We now emphasize this more clearly in the Discussion and explicitly note this differential responsiveness as a limitation in evaluating clocks for general use as surrogate endpoints. Future work will need to stratify and validate clocks in diverse populations to determine context-specific utility.

- Remarkably, Omicmage and DNAmEMRAge are put forward as 'prominent DNAm markers'. The catchy term GenX is reserved for them. Since they are relatively new and the code to produce them is not even available, this is simply impossible. These markers show particularly underwhelming associations with interventions and yet the authors in their discussion claim that they are promising. It is difficult not to link this to the conflicts of interest the authors stated.

To clarify: The term Gen X is ***not*** *reserved for OMICmAge or DNAmEMRAge. Rather, it refers broadly to interpretable DNAm biomarkers that provide system-level or mechanistic insight, such as inflammation, immune, brain, and metabolic system scores, as well as causal clocks like DamAge and AdaptAge. While OMICmAge and DNAmEMRAge are composite models that include such components, they themselves are not classified as Gen X clocks in our analysis.*

Their inclusion in the study is not based on a claim of superior performance, but because they represent an emerging direction in biomarker development focused on mechanistic interpretability. We report their responsiveness transparently and do not overstate their associations. As the reviewer points out, it is very clear from our figure that they do not show large aggregate changes. Our discussion highlights the conceptual value of interpretability, not raw effect size, as an important dimension in the future utility of DNAm biomarkers.

- The manuscript can be condensed significantly by increasing the information density and focusing on main analyses.

Thank you for the suggestion. We are happy to streamline the manuscript where appropriate, but

we would appreciate clarification on what you consider the "main analyses." Given the multi-layered structure of our study spanning biomarker responsiveness, intervention classes, population stratification, and mechanistic insights fit is important for us to understand which components you see as central versus secondary.

We would also respectfully note that this suggestion appears to conflict with your earlier comment characterizing the analyses as "ad hoc and difficult to interpret." That critique implied a lack of clear analytic hierarchy, whereas the current comment presumes a defined set of primary analyses. We welcome any further guidance you can provide to reconcile these two perspectives and to ensure the manuscript structure aligns with expectations.

- Are the authors aware of an intervention where participants were guided to quit smoking? It will be of great interest to see the effects on clocks that are designed to also predict smoking status (e.g Grimage).

Yes, we have already included such an intervention in our analysis. Specifically, a smoking cessation study is included in TranslAGE-Response and is discussed in detail in Figure 5B and 5C, where we stratify effects by population health status. This study provides a valuable case to examine the responsiveness of DNAm biomarkers, including those with components trained to predict smoking exposure, such as GrimAge and GrimAgeV2.

We have also provided the full list of associations between this intervention and all 110 DNAm biomarkers, including the prominent clocks and individual component scores, in Supplementary Table 14. We agree that this example is of particular relevance, and appreciate your interest in it.

- Y other biomarkers. What is Y?

Thank you for pointing this out. The phrase "Y other biomarkers" was a typographical error and has been corrected in the manuscript.

- The first figure discussed is figure 2 (not 1).

Figure 1 is referenced in the Introduction, where we introduce the conceptual framework motivating this study. The flow of the manuscript intentionally begins with Figure 1 to orient readers to the logic of DNAm biomarker evaluation before moving into the empirical results.

- There is a mismatch with the content of figure 1a and the results section describing the figure. Figures 1b and 1c are note even discussed. The purpose of the figures and the interpretation is debatable.

Figure 1A is discussed in the Introduction, where it is used to establish the conceptual framework for evaluating DNAm biomarkers along three axes: prognostic value, longitudinal stability, and interventional responsiveness. This framing is central to the study and precedes any empirical results by design.

Furthermore, Figures 1B and 1C are not omitted, they serve distinct illustrative purposes and are directly relevant to the manuscript's logic. Figure 1B represents the conventional approach of analyzing single biomarkers or single interventions in isolation, while Figure 1C presents our proposed integrative framework, which evaluates the full matrix of biomarkers and interventions. These figures are meant to clarify the motivation and novelty of our approach, not to display results.

We have now added brief clarifying statements in the Introduction to ensure their roles are unmistakable, but we stand by their inclusion as essential to understanding the study's design and contribution.

- The legend of figure 3 is of low quality.

This has been corrected

- Figure 4e shows more consistency among the dietary interventions then the (more noisy) pharmacological ones, yet the authors conclude the opposite.

Thank you for your comment. However, we would like to clarify that we do not conclude that pharmacological interventions are more consistent than dietary interventions. Rather, we report that pharmacological interventions induce greater effect sizes on DNAm biomarkers on average. This is distinct from consistency, which refers to within- and across-study reproducibility.

As you correctly point out, Figure 4E shows that dietary interventions may yield more consistent effects across biomarkers, albeit with smaller magnitudes. We have clarified this distinction between magnitude and consistency in the Discussion to avoid any confusion.

"It should be noted that pharmacological interventions tend to produce larger average effect sizes on DNAm biomarkers, whereas dietary interventions show greater cross-biomarker consistency, emphasizing that effect magnitude and consistency capture distinct and complementary dimensions of biomarker response"

- Suppl fig 1: figure content and legend do not match.

Thank you for pointing this out. The mismatch between the content and legend of Supplementary Figure 1 has been corrected.

Reviewer #3 (Remarks to the Author):

It is still under debate whether interventions impact the epigenetic aging process. The study of Sehgal et al. therefore addresses a very important and timely topic. It provides a comprehensive collection of 51 interventional studies and tested 16 prominent epigenetic clocks into the TranslAGE-Reponse database. Notably, several second generation clocks have coherent response, particularly to pharmacological interventions. While such a comprehensive comparison is intriguing, there are conceptional difficulties in numerical comparison of the number of studies - with very different designs, overlapping epigenetic biomarkers, and possible confounding changes in clinical parameters. Thus, I feel that the findings of this comparison (which of course could not be validated on independent sets of studies) should be more critically discussed. I have the following specific concerns:

1) Unfortunately, TranslAGE is apparently not easily accessible to other researchers. Furthermore, about half of the studies were generated by TrueDiagnostics and are apparently not open access. The authors should better clarify how they “intend for TranslAGE to become available as a resource to the geroscience community.”

Thank you for raising this important point. We completely agree that accessibility and transparency are essential for maximizing the utility of TranslAGE as a resource for the geroscience community.

To clarify: all publicly available datasets used in TranslAGE will be made fully accessible upon publication, including processed beta value files and standardized metadata (e.g., age, sex, treatment group, timepoint). We have already started this process by making a major part of the data available via our website: https://www.translage.io/responsive. For datasets contributed by TruDiagnostic, which are not currently public due to participant consent constraints, we are working with the original data custodians to define a compliant data-sharing pathway. Harmonized datasets will be returned to TruDiagnostic so that anyone may apply for them through TruDiagnostic. In the meantime, summary statistics and aggregated analyses from these studies will be included in the public release.

We are also preparing a dedicated data repository (e.g., via Zenodo or similar) along with all code used for processing, analysis, and figure generation, so that others can reproduce and build upon this work. These details have now been clarified in the updated Data Availability section

2) If 110 DNAm biomarkers are tested, additional adjustment for multiple testing may be required (particularly if the threshold is only set to 0.05).

In the revised manuscript, we have applied an independent testing framework (Li et al., 2011). Specifically, for each analysis we estimate the effective number of independent tests using PCA of the biomarker correlation structure, retaining components that explain 90% of the variance.

This ensures that multiple testing correction reflects the true dimensionality of the data, rather than assuming independence across all biomarkers.

3) Different types of interventional studies have been pooled – with different age ranges, treatment regimen, duration, etc. I understand that pooling of effect sizes may give clearer results, but from a biological perspective this may be a bit irritating. I would suggest to rather not show this.

Thank you for this thoughtful comment. We fully understand the concern that pooling across diverse interventional studies, with varying age ranges, treatment durations, and modalities, can obscure biological specificity. Our intention in presenting pooled effect size distributions was not to imply biological equivalence between interventions, but rather to provide a broad comparative overview of DNAm biomarker responsiveness across the field.

To address this concern, we have clarified in the text that this pooled analysis serves a descriptive purpose, to identify global trends and prioritize biomarkers that show consistent responsiveness across heterogeneous contexts. (Example from the discussion)

"Limitations of this study should be acknowledged. First, we pooled effect sizes across heterogeneous interventional studies that differed substantially in participant age ranges, intervention modalities, durations, and study designs. While this heterogeneity limits biological specificity and precludes interpretation of pooled effects as mechanistic equivalence, our intent was descriptive rather than causal: to characterize broad patterns of DNAm biomarker responsiveness and prioritize clocks that show consistent sensitivity to change. Accordingly, biological interpretation is emphasized within intervention classes, which are analyzed separately throughout the manuscript."

We also emphasize throughout the manuscript that meaningful biological interpretation should be made within intervention classes (e.g., pharmacological vs. lifestyle), which are analyzed separately in multiple figures (e.g., Figures 3, 4, and 5).

Nonetheless, we appreciate your point and have adjusted the framing of this analysis to avoid overinterpretation. We've also added clearer language distinguishing exploratory pooling from biologically driven subgroup comparisons.

4) The different DNAm biomarkers are not independent. There is probably a significant overlap in the CpGs between these signatures that needs to be specified and discussed.

We agree with the reviewer that DNAm biomarkers are not independent, as many share overlapping CpG sites. We had previously acknowledged this non-independence as the rationale for using a nominal p-value threshold of 0.05. In the revised manuscript, we now formalize this

approach by applying an independent testing framework (Li et al., 2011). Specifically, for each analysis we estimate the effective number of independent tests using PCA of the biomarker correlation structure, retaining components that explain 90% of the variance. This ensures that multiple testing correction reflects the true dimensionality of the data, rather than assuming independence across all biomarkers.

5) Gen 2+ clocks have been trained based on additional age-associated parameters. While I fully agree that this may be valuable to quantify aspects of biological aging, there is also the danger that effects are largely attributed to changes in these parameters (e.g. to cellular composition or CRP). For example. GrimAgeV2 was also trained on c-reactive protein – a relevant parameter associated with effect size (Fig 6). Yet, interventions that change CRP do not necessarily impact the aging process but rather signify health status and inflammation (PMID: 38692281). Thus, caution needs to be taken when using Gen 2+ clocks to investigate effects of interventions on the aging process. I believe this is a general conceptual problem that needs to be discussed.

Thank you for raising this important and nuanced point. We fully agree that Generation 2+ clocks, including GrimAgeV2, were trained on composite age-related risk factors, such as C-reactive protein (CRP), smoking history, or mortality risk, and that this introduces complexity when interpreting their responsiveness. Specifically, observed changes in these clocks may reflect shifts in intermediate health markers (e.g., inflammation) rather than direct modification of the aging process itself.

We view this not as a flaw, but as a critical interpretive boundary. As you rightly note, interventions that reduce CRP may reflect improvements in immune regulation or acute health status rather than broader impacts on aging trajectories. However, given that systemic inflammation is a known contributor to aging biology, we believe these clocks remain valuable for capturing relevant, and potentially reversible, dimensions of biological aging.

That said, we have added language in the Discussion to explicitly caution against over-interpreting reductions in Gen 2+ clocks as definitive anti-aging effects, especially when driven by mutable parameters like CRP. We also emphasize the need for future studies to triangulate DNAm biomarker changes with functional and clinical outcomes to distinguish health modulation from aging modulation.

*“**Limitations of this study should be acknowledged.** First, we pooled effect sizes across heterogeneous interventional studies that differed substantially in participant age ranges, intervention modalities, durations, and study designs. While this heterogeneity limits biological specificity and precludes interpretation of pooled effects as mechanistic equivalence, our intent was descriptive rather than causal: to characterize broad patterns of DNAm biomarker responsiveness and prioritize clocks that show consistent sensitivity to change. Accordingly, biological interpretation is emphasized within intervention classes, which are analyzed separately throughout the manuscript. Second, variability in study quality, sample size, and intervention relevance is an inherent limitation; however, this diversity provides a wide distribution of effect sizes that is informative for benchmarking biomarker behavior rather than evaluating individual intervention efficacy. Third, lack of harmonized preprocessing and batch correction across studies*

may affect comparability, and future updates to TranslAGE-Response will incorporate such adjustments where metadata permit. ***Fourth, interpretation of Generation 2+ clocks requires caution, as their training on composite risk factors (e.g., C-reactive protein in GrimAgeV2) means observed responsiveness may reflect modulation of intermediate health or inflammatory states rather than direct effects on aging biology per se.*** *Fifth, the absence of an empirically defined minimal clinically important difference (MCID) for DNAm clocks remains a key barrier to their use as surrogate endpoints; future work must anchor clock changes to clinically meaningful functional, physiological, or disease-specific outcomes. Finally, although standardized effect sizes may appear modest, for widely used clocks such as Horvath, Hannum, PhenoAge, and GrimAge, where the standard deviation of epigenetic age deviation is typically on the order of ~4–6 years, an effect size of ~0.2 corresponds to an approximate one-year shift in epigenetic age, indicating that the observed changes are not biologically trivial."*

Thank you for highlighting this conceptual limitation, we agree it deserves attention.

Minor:

6) It would be good to mention the total number of samples analyzed. Furthermore, additional information on the study design of the 51 studies should be provided in the supplemental table (particularly for those studies that are apparently not yet published). Please include also links to the publications, if available.

We appreciate the reviewer's suggestion. The total number of samples analyzed, study design details for all 51 datasets (totaling 3128 samples), as well as GEO/EMTAB accessions, clinical trial identifiers, and publication links (where available) are provided in Supplementary Table 1. For example the following columns are added in Supplementary Table 1: Intervention, GEO ID or Dataset ID, , Link to main publication (PMID), Sample Type. Array.Study ID (Or study info source).Study Design: Primary Purpose. Study Design: Allocation. Study Design: Interventional Model. Study Design: Masking, Inclusion/ Exclusion Criteria, Intervention Regimen, MoA, Intervention Category, Condition, Sex Distribution, Sample count, Age Range, Length of intervention(tmax) and more. Additionally all of this data is now available at translage.io.

7) P3: "The first epigenetic clocks" are not cited here (perhaps rephrase, "relatively early epigenetic clocks".)

Thank you for this suggestion. Could you clarify which specific clocks you are referring to as "the first epigenetic clocks"? For example, do you mean Horvath's pan-tissue clock and Hannum's blood-based clock, or a broader set of early-generation predictors? This will help us phrase it more precisely (e.g., "relatively early epigenetic clocks") in the revision.

8) P4: Please include a reference that these clocks are "… collectively known as Generation 2+ clocks". I believe they are more commonly referred to as second generation clocks.

We thank the reviewer for this comment. While "second-generation clocks" is indeed a commonly used term, we have deliberately adopted the terminology "Generation 2+ clocks" to reflect a broader and more accurate classification. The existing "first-" vs. "second-generation" framing oversimplifies the landscape of clocks, which now extend beyond simple two-generation boundaries. Our use of "Generation 2+" is meant to distinguish this newer class from the earliest clocks, while acknowledging the diversity of methods and features encompassed by more recent models.

9) Last section of the introduction "… and Y other DNAm biomarkers".

We thank the reviewer for pointing this out. We have corrected the last section of the introduction to read "… and 92 other DNAm biomarkers."

10) Are there differences by sex? And of the other clinical covariates (as also suggested in the comment that still remained in the word file)

We thank the reviewer for this suggestion. We have partly addressed this in Figure 5A, which shows the association of the clock with sex. In addition, we examined associations with seven other clinical covariates, which are also presented in Figure 5A. We are preparing a separate manuscript on sex-stratified analyses, especially for diet meta-analyses where there are enough studies to perform stratified analyses with sufficient power (see Reviewer 1 comments for more details).

11) In figure 3A (label in the legend is wrong) is the standard deviation indicated? Could it be that the effect size is largely affected by few individual studies?

We thank the reviewer for this observation. The standard deviation is indicated in Figure 3A, and we have corrected the legend label to make this clearer. While variability between studies is present, the overall effect size is not driven by just a few individual studies.

12) The effect size is presumably very small. If effects on epigenetic age predictions are only marginal (i.e. few days), then this should be discussed.

We thank the reviewer for this comment. While the reported results are presented as standardized effect sizes and therefore do not directly correspond to changes in years of epigenetic age deviation, it is important to note that the standard deviation of age deviation for most DNAm clocks is on the order of ~5 years, although this varies by biomarker. Under this scaling, a standardized effect size of 0.2 corresponds to an approximate 1-year shift in epigenetic age. Several of the dietary and exercise interventions evaluated in this study fall within this range, indicating that the

observed effects are not merely marginal when interpreted on an absolute age scale. We have added this clarification to the Discussion to aid interpretation of effect size magnitude.

13) I am not sure if it has been shown that DNAm biomarkers preferentially capture stochastic epigenetic noise – while this is conceivable and supported by some models, it is still not proven. Furthermore, their finding on this aspect was only shown for StocH vs StocP and no other models.

We thank the reviewer for this comment. Our analysis focused on the models currently available in the literature. The observed preference for stochastic epigenetic noise was shown specifically for StocH vs. StocP, and while this does not generalize across all models, it provides an initial proof-of-concept. We believe it is worth highlighting as an idea that merits further exploration in future work.

14) The discussion repeats some of the results. Instead, it would be valuable to critically discuss the limitations of this study.

We thank the reviewer for this suggestion. We have updated the Discussion to reduce repetition of results and to include a more critical appraisal of the study's limitations.

"Limitations of this study should be acknowledged. First, we pooled effect sizes across heterogeneous interventional studies that differed substantially in participant age ranges, intervention modalities, durations, and study designs. While this heterogeneity limits biological specificity and precludes interpretation of pooled effects as mechanistic equivalence, our intent was descriptive rather than causal: to characterize broad patterns of DNAm biomarker responsiveness and prioritize clocks that show consistent sensitivity to change. Accordingly, biological interpretation is emphasized within intervention classes, which are analyzed separately throughout the manuscript. Second, variability in study quality, sample size, and intervention relevance is an inherent limitation; however, this diversity provides a wide distribution of effect sizes that is informative for benchmarking biomarker behavior rather than evaluating individual intervention efficacy. Third, lack of harmonized preprocessing and batch correction across studies may affect comparability, and future updates to TranslAGE-Response will incorporate such adjustments where metadata permit. Fourth, interpretation of Generation 2+ clocks requires caution, as their training on composite risk factors (e.g., C-reactive protein in GrimAgeV2) means observed responsiveness may reflect modulation of intermediate health or inflammatory states rather than direct effects on aging biology per se. Fifth, the absence of an empirically defined minimal clinically important difference (MCID) for DNAm clocks remains a key barrier to their use as surrogate endpoints; future work must anchor clock changes to clinically meaningful functional, physiological, or disease-specific outcomes. Finally, although standardized effect sizes may appear modest, for widely used clocks such as Horvath, Hannum, PhenoAge, and GrimAge, where the standard deviation of epigenetic age deviation is typically on the order of ~4–6 years, an effect size of ~0.2 corresponds to an approximate one-year shift in epigenetic age, indicating that the observed changes are not biologically trivial."

15) Are there systematic effects of interventions on cell count predictions?

We thank the reviewer for raising this point. In some cases, interventions do appear to have systematic effects on cell count predictions. We are working on an additional manuscript showing these effects, that being said, we examined the responsiveness of the clocks after regressing out cell counts, which is presented in the supplementary figures 1 and they do not show any major differences.

16) Please provide reference for MethylCIPHER 2.0

Thank you for the suggestion. MethylCIPHER 2.0 is available as an R package via the Higgins- Chen (HigginsChenLab) GitHub repository (forked from MorganLevineLab), though the formal peer-reviewed manuscript is still pending. We have cited the link to the updated version of Methylcipher in code availability.

17) Perhaps figure 4c might be represented as a heatmap?

We thank the reviewer for this helpful suggestion. We agree that a heatmap could be a useful way to represent the data. However, in this case we chose a stacked bar plot because it allows the relative quantification of agreement and disagreement to be conveyed more directly. We believe this format better highlights the comparative proportions across conditions, which was the focus of the analysis.

18) There are several typos and inconsistencies that should be corrected.

We thank the reviewer for pointing this out. We have carefully reviewed the manuscript and corrected typos and inconsistencies as much as possible. If any remain, we would greatly appreciate it if the reviewer could kindly highlight them so we can ensure they are fully addressed in the revision.

Editors' comments:

1. In line with the comments from the reviewers', we feel that there would need to be substantially more information provided on the different interventional studies, particularly with respect to those that are private or have not been previously published. For example, information should be made available regarding: when the interventions were conducted, whether the interventions were all prospectively designed, whether they are registered, whether they are single-arm or randomized, inclusion/exclusion criteria, number of participants, specifics as to the intervention including dose, and duration of the interventions. We would ask you to consider presenting the essential information for each interventional study in a table that would be more accessible to the reader. Please also clarify how these interventional studies were selected for inclusion in the manuscript.

We thank the editor for this important suggestion. We have now expanded Supplementary Table 1 to include additional information for each interventional study, covering details such as registration status (clinical trial identifiers where available), design (single-arm vs. randomized), participant numbers, inclusion/exclusion criteria when reported, intervention specifics (dose and duration), and timing of the interventions. For studies that are unpublished or private, we have provided all available information and clearly indicated when details could not be obtained.

We have also clarified in the Methods section how interventional studies were selected for inclusion in the manuscript. In brief, we included all studies for which DNA methylation data were publicly available in repositories such as GEO or ArrayExpress, or which were made available to us by collaborators, provided they met our quality control thresholds.

We agree that presenting these details in a structured format improves accessibility, and we believe the revised table addresses this concern.

2. Similarly to the reviewers, we feel that it would be important that the TranslAGE database be made publicly available, as we feel that much of the value of the work comes from providing these data to the community.

We thank the reviewers for this valuable suggestion. In response, we have made a version of the TranslAGE (translage.io) available through an interactive dashboard. This provides access to the curated datasets and summary statistics, making the resource more accessible to the community while respecting data use agreements.

3. It does not seem completely clear to what extent information on the OMICmAge, DNAmEMRage, SystemsAge clocks is publicly available, and please ensure that citations for these clocks is provided. If the underlying details of these clocks aren't available, we would ask that complete information on these clocks be made publicly available.

We thank the reviewers for this important point. SystemsAge and all other major clocks are fully available through the methylCIPHER R package, and we have provided the appropriate citation in the manuscript. The other two clocks, OMICmAge and DNAmEMRage, are proprietary algorithms. These are available commercially through TruDiagnostic, and we have clarified this in the text and ensured that citations are included.

4. Please ensure that titles/legends are provided for all Supplementary Tables.

We thank the reviewer for this note. Titles and legends have now been provided for all Supplementary Tables to ensure clarity and ease of reference.

5. By our estimation, the text (Introduction, Results and Discussion) is currently 6,436 words, which is substantially over the limit of 4,000 words. We have some flexibility on this, but would ask you to shorten the manuscript to as close to 5,000 words as possible

We thank the editors for pointing this out. We have revised the manuscript to reduce redundancy and streamline the Introduction, Results, and Discussion.

.

Reviewers' Comments:

Reviewer #1 (Remarks to the Author):

I thank the authors for thoroughly addressing my comments. I have no further comments. Congratulations on this important study.

We thank the reviewer for evaluating our manuscript and we are glad we were able to address all of their comments!

Reviewer #2 (Remarks to the Author):

While it was obvious from my first review that I am less enthusiastic about the work than the other reviewers and editor, I appreciate the authors' responsiveness, effort to make the results/data publicly available (as I understand this process is ongoing), and the careful additions to the discussion. Two questions remain for me:

We are glad that we were able to address a majority of the reviewers' concerns. We would be happy to address the remaining questions the reviewer has as well.

1. The authors discuss correction for multiple testing in their rebuttal and manuscript, but do not actually implement it. The authors argue (in line with my original remark) that taking the correlation between tests into account (instead of performing Bonferroni correction) is a way forward. I also agree that taking the number of the principal components capturing 90% of the total variance may be defendable. However, the authors continue to state that the use (and also report) on findings $P<0.05$. I believe this should be changed and only findings significant after accounting for multiple testing should be reported to prevent over-interpretation with respect to the utility of epigenetic clocks. (On the side: I still do not understand why T tests instead of multivariable analyses were done to account for the obvious influence of covariates)

We appreciate the reviewer being rigorous in statistical significance of the clocks. As the reviewer pointed out, inspired by their suggested method we used the independent testing framework by Li et al. We detailed out the method for this as well as how many independent features were used in each analysis in Supplementary Table 20.

For each analysis where such multiple testing was performed we report both the nominal results and the results after multiple testing correction. Importantly, the interpretation of the findings in the manuscript is based on the results that remain significant after correction. Nominal $P < 0.05$ values are presented only for transparency so that readers can clearly see the magnitude and distribution of effects across clocks. Furthermore, providing nominal p-values allows other researchers to take our p-values and perform alternative methods of multiple testing correction if they wish to investigate the results if a different method were to be used.

For example, in Figure 4B we clearly annotate clocks that are significant before and after multiple testing with the following text in the legend:
"Scatter plot of effect sizes of interventions on DNAm biomarkers where each point is one intervention study: Significant effect sizes are marked with red asterisks and blue hashtags (* p < 0.05, ** p < 0.01, *** p < 0.001, # significant after multiple testing corrections)."

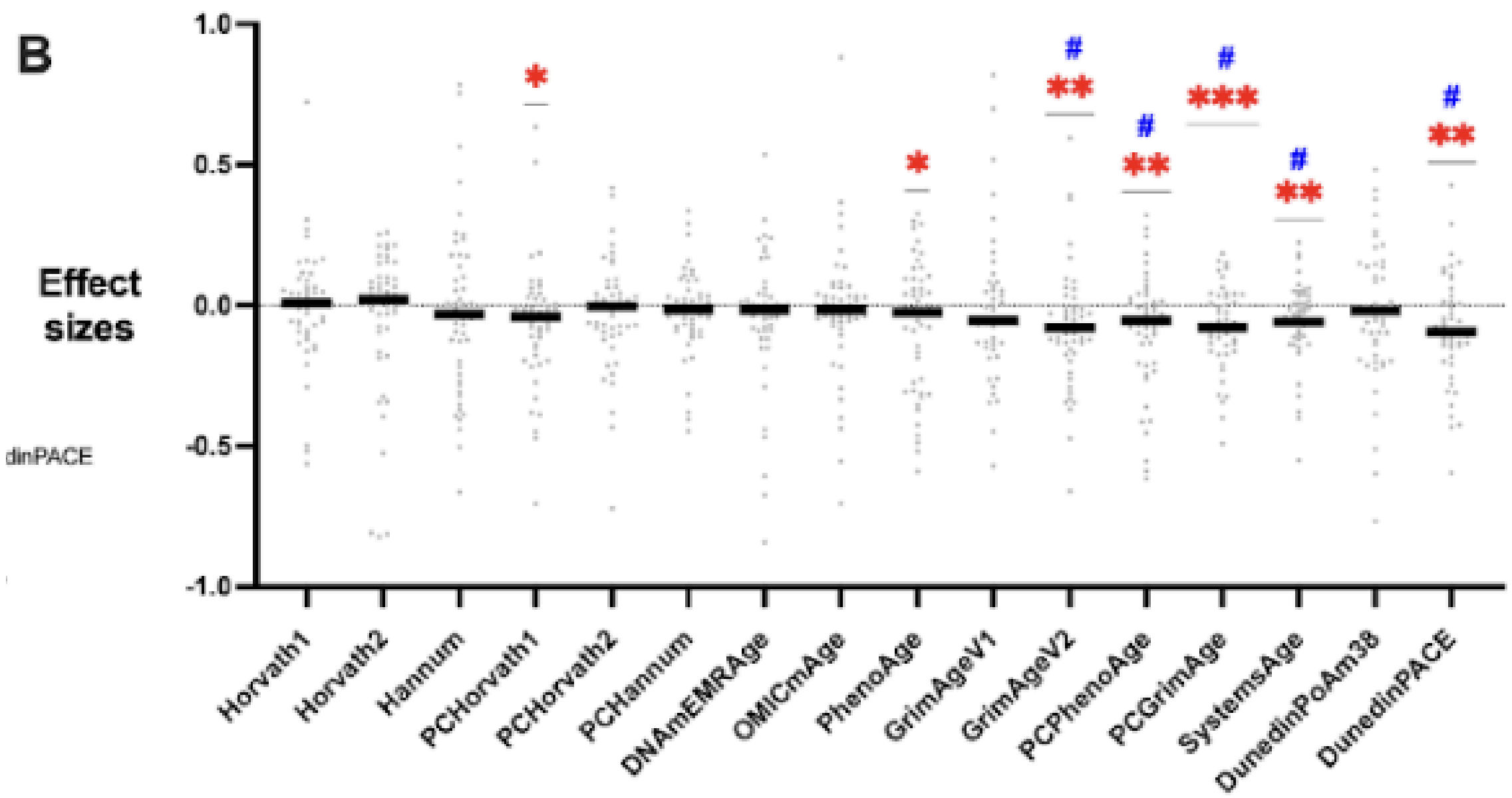

The corresponding text in the Results also explicitly distinguishes between clocks that remain significant after correction and those that do not "This analysis revealed that all Gen 2+ Reliable biomarkers showed significant decreases. These include GrimAgeV2 (mean = -0.0814, p= 0.0075), PCPhenoAge (mean = -0.08876, p = 0.003), PCGrimAge (mean = -0.07843, p =0.0003), SystemsAge (mean = -0.05859, p = 0.0043) and DunedinPACE which had the largest decrease (mean = -0.0891, p = 0.0012). **(Significant after multiple testing correction)** In contrast, only a single Gen 1 (reliable) clock significantly decreased across all interventions (PCHorvath1 mean = -0.06674, P-value = 0.029), and a single Gen 2 non-reliable DNAm biomarker decreased (PhenoAge mean = -0.06735, p = 0.0408). **(Not significant after multiple testing correction)**"

With respect to the reviewer's comment regarding the use of t tests rather than multivariable models, our primary goal was to evaluate the average intervention response of DNAm biomarkers across a heterogeneous set of studies. Because individual-level covariate data were not consistently available across all intervention datasets, fitting a unified multivariable model across studies was not feasible without substantially reducing the number of studies included or applying inconsistent adjustment strategies.

To assess the potential influence of covariates, we conducted an additional analysis examining whether study characteristics influenced DNAm biomarker responses to interventions (Figure 5). Specifically, we evaluated associations between intervention effect sizes and study-level

variables including sample size, intervention duration, mean age, age distribution (SD, minimum, and range), and percent female. The results, summarized as Z-scores in Figure 5, indicate that intervention responses were largely independent of these study characteristics.

Taken together, these analyses suggest that the observed intervention-associated changes in DNAm biomarkers are robust and not primarily driven by variation in demographic composition or study design across studies.

2. The authors state that 'The other two clocks, OMICmAge and DNAmEMRage, are proprietary algorithms. These are available commercially through TruDiagnostic, and we have clarified this in the text and ensured that citations are included.' I strongly believe this should have the consequence that both clocks cannot be included in the paper. It goes against open science and reproducibility. The clock-field already suffers from potential conflicts of interests (which for clinical studies are well-known to influence outcomes of scientific studies). The fact that the clocks cannot be used by the field turn a scientific paper into an advertisement of a commercial product.

We thank the reviewer for raising this important concern regarding openness and reproducibility. We agree that transparency and reproducibility are essential for the advancement of the aging biomarker field.

Importantly, OMICmAge has now been fully described in a peer-reviewed publication in Nature Aging (OMICmAge quantifies biological age by integrating multi-omics with electronic medical records, Chen et al). The R code used to develop and validate EMRAge, DNAmEMRAge, and OMICmAge is publicly available at https://github.com/LaskySuLab/OMICmAge/, which is the GitHub repository of an academic lab. In addition, code to calculate the metrics is accessible through TruDiagnostic's DNAm Analysis Software. Access to this software can be requested at https://www.trudiagnostic.com/softwarerequest/ at no cost for academic research purposes under a standard noncommercial license agreement. This allows researchers to apply the clocks for academic and nonprofit research while protecting the commercial implementation. This established process has already allowed over 10 academic research groups to access the algorithms.

We have decided to add Jessica Lasky-Su as an author to the current manuscript. We have already been collaborating extensively with her to build out the broader TranslAGE framework including translage.io and the future directions of the platform. Furthermore, she developed OMICmAge and DNAmEMRAge in collaboration with TruDiagnostic. Thus, she meets criteria to be an author. Dr. Lasky-Su, as the academic developer of OMICmAge and DNAmEMRAge, certifies she is committed to ensuring these algorithms are readily available and remain readily available for academic and research use.

Our intention in including OMICmAge and DNAmEMRAge was not to promote proprietary tools but to provide a comprehensive benchmarking comparison across a broad range of DNA

methylation biomarkers currently used in the field. Importantly, the inclusion of these clocks does not drive the conclusions of the manuscript. In fact, both OMICmAge and DNAmEMRAge showed little to no responsiveness to interventions in our analyses and did not demonstrate statistically significant changes across the intervention datasets evaluated. As a result, their inclusion does not present these clocks in a favorable light and therefore cannot reasonably be interpreted as promotional.

More broadly, the main findings of the manuscript remain unchanged when analyses are restricted to fully open and publicly available clocks. We have clarified the availability of code and software access in the manuscript and ensured that the relevant citations are included so that readers understand how these models can be accessed and reproduced in academic settings.

Our new code availability statement:

"Code to calculate all clocks (except for OMICmAge and DNAmEMRAge) are available at https://github.com/HigginsChenLab/methylCIPHER. Code to calculate OMICmAge, DNAmEMRAge, and the associated underlying algorithm can be requested at https://www.trudiagnostic.com/softwarerequest/, at no cost for research purposes. Access is contingent upon the requestor agreeing to a standard, noncommercial software license agreement that confirms the intended use is strictly for academic, nonprofit research and that the requestor will not attempt to reverse engineer or commercially exploit the software. All R codes used to develop and validate EMRAge, DNAmEMRAge and OMICmAge are available at https://github.com/LaskySuLab/OMICmAge/."

Reviewer #3 (Remarks to the Author):

As indicated previously, this integrative analysis of 51 interventional studies provides an important and timely overview of aging biomarkers. In particular, the observed deviations in predictions are highly informative for the field, as they help delineate the current strength and limitations of epigenetic clocks. I appreciate that the revised manuscript now includes a more explicit discussion of these limitations, especially the point that generation 2+ clocks may primarily capture health-related parameters rather than the aging trajectory per se. While such measures are undoubtedly relevant for health outcomes, they should not be equated with biological rejuvenation.

Overall, the authors have carefully revised the manuscript and addressed the majority of my concerns. Although not all suggestions were fully implemented, their explanations are reasonable and scientifically ok. The authors also state that all publicly available datasets used in TranslAGE will be fully accessible upon publication and that they are working toward a compliant data-sharing pathway for the TruDiagnostic datasets. I assume that this will be finalized prior to publication. In this regard, Supplementary Table 1 is already a helpful step toward greater transparency.

We thank the reviewer for their thoughtful evaluation and for recognizing the value of this integrative analysis. We appreciate the positive feedback and the acknowledgment that the manuscript now more clearly discusses the strengths and limitations of current epigenetic clocks. We agree that while many generation 2+ clocks capture health-related physiological risk signals, this should not be directly interpreted as evidence of biological rejuvenation. The manuscript therefore explicitly distinguishes between biomarkers that reflect health status and those that may more directly capture aspects of the biological aging process.

We also appreciate the reviewer's recognition of the efforts to improve transparency and data availability. As stated in the manuscript, all publicly available datasets used in TranslAGE will be fully accessible upon publication. In parallel, we are working to establish a compliant data-sharing pathway for the TruDiagnostic datasets that respects participant privacy and institutional data governance requirements. Our goal is to finalize this pathway prior to publication to ensure that the analyses can be reproduced by the research community.

Finally, we are glad that Supplementary Table 1 was helpful in improving transparency regarding dataset sources. We appreciate the reviewer's constructive feedback throughout the review process, which has helped strengthen the manuscript.

Editors comments

We thank the editors for the careful evaluation of the manuscript and for highlighting these important issues. We respond to each point below.

1. Please respond in full to referee #2's first concern regarding multiple testing, which we feel would be important for the rigor of the analysis.

We agree that appropriate correction for multiple testing is essential for the rigor of the analysis. Following the reviewer's recommendation, we implemented an independent testing framework based on the method of Li et al., which accounts for the correlation structure among the DNAm biomarkers rather than assuming independence as in Bonferroni correction. Specifically, we estimated the effective number of independent tests by performing principal component analysis of the biomarker correlation matrix and using the number of principal components required to explain 90% of the total variance.

The resulting effective number of tests was then used to determine the corrected significance thresholds. The full methodology and the number of independent tests used for each analysis are now detailed in Supplementary Table 20.

In the manuscript, we clearly distinguish between nominal significance ($P < 0.05$) and results that remain significant after multiple testing corrections. Interpretation of the findings is based on the corrected results, while nominal P values are presented only to provide transparency

regarding the distribution and magnitude of effects across biomarkers. Furthermore, providing nominal p-values allows other researchers to take our p-values and perform alternative methods of multiple testing correction if they wish to investigate the results if a different method were to be used.

2. Both referees #2 and #3 feel that public availability of the data would be important, and from an editorial point of view, we see this as an essential aspect of the interest of publishing this type of analysis and resource. We therefore feel that publication would need to be contingent on providing a path for access to the data to researchers, with as much as possible of the data being freely available, and if necessary the remainder being available through controlled access. In this respect, it seems somewhat unclear what type of data would be available in the TranslAGE web site for each of the different trials, and we ask that a more granular description be provided, as well as more details as to the status of each of the 51 trials in Supp. Table 1 (this might be best shown in a separate column, indicating the status of data availability for each of the trials). Again, for this type of study, we feel that the more data that can be made freely available the better, and that a clear path to data access needs to be provided for all of the data.

We fully agree that data accessibility is critical for a resource of this type. Our goal is to maximize openness while respecting the governance and privacy constraints associated with several of the contributing studies.

All publicly available datasets used in TranslAGE are now freely accessible through the TrasnlAGE dashboard (a short video for description is now provided in TrasnlAGE data availability), and we will provide direct links and accession identifiers through the TranslAGE website. For each of the 51 intervention studies included in the analysis, we will provide a clear description of the data availability status.

To improve transparency, Supplementary Table 1 now includes an additional column called "Access" indicating the data availability pathway for each trial. Specifically, datasets are categorized as:

- *Publicly available datasets, which can be accessed directly through public repositories (e.g., GEO, ArrayExpress, or other open databases). Downloadable links and data is provided.*

- *Controlled-access datasets, where data are available through formal application to the corresponding data access committees or institutional repositories. A link to fill such forms is provided.*

- *Partner datasets, such as those generated through TruDiagnostic collaborations, which cannot be publicly released at the individual-level due to participant consent and institutional restrictions but for which we are establishing a controlled-access pathway. A link to fill research partnership form is provided.*

In addition, the TranslAGE website will provide, for each trial, summary-level results including intervention effect sizes, statistical metrics, study metadata (e.g., intervention type, duration, sample size, demographics), and the derived biomarker response metrics used in the analyses. This structure will allow researchers to explore the full benchmarking results while ensuring that individual-level participant data are accessed through appropriate governance mechanisms where required.

3. With regard to the OMICmAge and DNAmEMRage clocks, which are proprietary, we ask that you clarify what type of detailed information on these clocks has been previously published (or is contained in the current manuscript), that would allow researchers to understand how these clocks work and can be used. In line with referee #2's second point, we would encourage that the code underlying these clocks be made publicly available in the interest of transparency and reproducibility.

We agree that transparency around the clocks included in the analysis is important. OMICmAge has been described in a recent peer-reviewed publication (Nature Aging), where methodological details and code availability are provided. In particular, the R code used to develop and validate EMRAge, DNAmEMRAge, and OMICmAge is publicly available at:

https://github.com/LaskySuLab/OMICmAge/

In addition, the implementation used to calculate these metrics is available through TruDiagnostic's DNAm Analysis Software. Access to this software can be requested at no cost for academic research purposes through a standard noncommercial license agreement (https://www.trudiagnostic.com/softwarerequest/). This allows academic researchers to compute the metrics while protecting the commercial deployment of the software.

We also note that the inclusion of these clocks does not influence the conclusions of the study. In our analyses, neither OMICmAge nor DNAmEMRAge demonstrated consistent responsiveness to interventions across the evaluated studies, and they did not show statistically significant intervention-associated changes. Their inclusion therefore does not strengthen the primary claims of the manuscript and cannot reasonably be interpreted as promotional.

We have clarified these points in the manuscript and ensured that the relevant publications and code availability information are cited.

We appreciate the editors' guidance on these issues and believe these clarifications strengthen the rigor, transparency, and utility of the TranslAGE resource for the research community.
